# Assessment of Meltblown High-Temperature Polymers for Fluorine-Free Low-Grammage Nonwoven Structures

**DOI:** 10.3390/polym18212684

**Published:** 2026-10-29

**Authors:** Tim Hiller, Simon Ringger, Marc Philip Vocht, Mehdi Azimian

**Affiliations:** German Institutes of Textile and Fiber Research Denkendorf, Koerschtal Str. 26, 73770 Denkendorf, Germany; simon.ringger@ditf.de (S.R.); mehdi.azimian@ditf.de (M.A.)

**Keywords:** high-performance polymers, PFAS alternatives, fibers, nonwovens, spunmelt, meltblow, thermal resistance, reinforcement structures, high-temperature applications, PFAS replacement

## Abstract

The proposed restriction of per- and polyfluoroalkyl substances (PFAS) by the European Chemicals Agency (ECHA) covers over 10,000 different substances. The affected materials also include PFAS-related fluoropolymers such as polytetrafluoroethylene (PTFE). Thus, the identification and establishment of substitutes—not only of materials, but also by alternative processing technologies—is not only environmentally desirable, but also an extremely pressing economic issue. This study deals with the potential to process high-temperature polymers into thin-layer-nonwovens. In this context, the meltblow processing of polyethersulfone (PESU) as an amorphous polymer from melt is demonstrated for the first time to the best of the authors’ knowledge. The resulting fabrics were compared with polyetheretherketone (PEEK) as a high-performance reference material owing to its excellent chemical resistance and mechanical properties, as well as to polyphenylene sulfide (PPS), the more widely used industrial standard because of its lower cost, though it exhibits a lower thermal stability, chemical resistance, and mechanical performance than PEEK. Fabrics of 10 g m^−^^2^ were successfully produced with median fiber diameters of 1.39 µm to 2.29 µm. PESU together with PPS achieved the finest median fiber diameters; however, PESU exhibited a lower tensile strength and substantially higher residual elongation than both PEEK and PPS, indicating the need for post-processing by calendering.

## 1. Introduction

In 2023, the European Chemicals Agency (ECHA) received a proposal for a broad restriction of per- and polyfluoroalkyl substances (PFAS) in the European Union (EU) under the REACH regulation [1]. The proposed restriction may affect over 10,000 different substances and is proclaimed to be the “most comprehensive restriction proposal to date under REACH” (Registration, Evaluation, Authorization and Restriction of Chemicals, Regulation (EU) No 1907/2006). Due to the stability of carbon–fluorine bonds, per- and polyfluoroalkyl substances (PFAS) are well-known for their thermal and chemical stability and widely used for these same reasons, especially in environments with high corrosion and temperature requirements [2]. Besides technical applications such as heat exchangers and membranes, they are also used in consumer goods such as water-repellent non-stick coatings [3] and food packaging [4]. For example, pure polytetrafluorethylene (PTFE, also known as Teflon©) retains excellent mechanical properties up to temperatures of 260 °C [5], and even up to 320 °C in composites [6]. However, the environmental persistence of many PFAS, in addition to their resistance to thermal and chemical degradation, is a major concern [7]. Therefore, they are known as “forever chemicals” [2]. The critical problem is that these long-lived substances can bioaccumulate in living organisms [8], which can potentially cause toxic effects [9]. In this context, studies show a correlation between exposure to PFAS and serious diseases such as cancer, thyroid diseases and immunological disorders [10]. Consequently, identifying and establishing suitable alternative materials has become both an environmental and an economic priority. This is particularly challenging in critical sectors such as medical technology and environmental applications, where substitute materials may not yet provide equivalent safety or performance [11]. High-performance industries using PFAS in components are also affected. Examples of this are membranes for lithium-ion batteries or fuel cells. PFAS-free alternatives are partially feasible, but not yet sufficiently established in the market [12].

Developing more sustainable polymer materials requires balancing environmental considerations with the performance needed for the intended application. Although reducing the use of PFAS including fluoropolymers like PTFE or PVDF is an important objective, potential alternatives must also provide adequate thermal stability, chemical resistance, and mechanical strength. Their suitability also depends on processing requirements, energy consumption, material costs, and compatibility with existing manufacturing technologies. Melt-processable high-temperature polymers are therefore of interest for applications requiring both thermal performance and efficient nonwoven production. However, their suitability as alternatives to fluorine-containing materials must be assessed specifically for each application.

The thermal stability of semicrystalline polymers depends on their glass transition temperature (*T_g_*) [13] and crystallite melting temperature (*T_m_*) [14] or softening temperature. Standard polymers as well as technical polymers cannot be used to replace PFAS due to their limited operating temperatures. Specifically, this includes the mass polymers polyethylene (PE) and polypropylene (PP), as well as polyesters and polyamides whose upper temperature limits in continuous use are typically below about 120 °C [13,14,15,16,17]. For this purpose, polymers with high temperature resistance—high-performance polymers (HPPs)—have been developed. These include polyimide (PI), polyetherimide (PEI) and polyetheretherketone (PEEK) [18] and offer the potential to replace fluoropolymers in selected applications. Common characteristics of high-temperature polymers are a high *T_g_*, a low *tan δ*-value and also a relatively low dielectric permittivity [19,20]. This is due to the aromatic or polyaromatic structures of the repeating units of these polymers, which restrict the rotation of the polymer chain [18]. However, for semicrystalline polymers, *T_m_*, the crystalline parts of the polymer melt is more relevant than *T_g_* [19], and, therefore, their crystallinity also determines their temperature resistance.

The meltblow process offers a high-throughput method to produce fine fibers in the diameter range of <1 µm to 30 µm [21], spun from the melt directly as a nonwoven fabric using a tangential stream of high-velocity hot air. The variability of typical base weights between 1 g m^−2^ to 400 g m^−2^ [22] allows the production of “thin” structures applicable in layer stacks or in composite materials. Here, they can function as a filtration medium, a carrier layer for other functional layers or a spacer, for example. A requirement for processing thermoplastics via meltblowing is the high flowability of the material, characterized by a low viscosity at its processing temperature [23]. However, this cannot be achieved with most of the high-temperature (HT) polymers, e.g., polyimides. Many high-performance polymers are not suitable for meltblow processing. This can be because they are not melt-processable in principle (e.g., degradation before melting), as is the case for most aromatic polyimides. Furthermore, polymers such as poly(arylether)sulfones (PAES), polyphenylsulfones (PPSU) and PEI exhibit melt viscosities that are too high for meltblowing, despite being melt-processable. Some of these polymers are spun from solution into fibers using wet- or dry-spinning processes [24] or deposited into nonwovens using the comparatively unproductive electrospinning process [25]. Among semi-crystalline high-performance polymers, PEEK and polyphenylene sulfide (PPS) have so far been identified as two polymer types providing both the combination of a melting temperature below 400 °C and sufficiently low melt viscosities to be processed directly from polymer melt into the fine(st) fibers to form a nonwoven using the productive meltblow process [26].

Beyond meltblow processing, PFAS alternatives are also being investigated based on standard polymers like polyethylene terephthalate (PET) and polylactic acid (PLA) as well as in natural materials (starch, chitosan, alginates, micro- and nanofibrillated cellulose), especially for use in laminates. While some of these materials exhibit suitable properties for specific requirements, their overall performance may still be inadequate [4]. PEEK offers a high temperature resistance (long-term) up to approximately 260 °C [27] and also excellent mechanical properties, flame-retardant properties, as well as high resistance to (UV-) radiation and chemicals [28]. In contrast to further polyaryletherketones (e.g., poly(phthalazinone ether ketone), PPEK), which partially permit even higher operating temperatures, the semi-crystalline areas of PEEK (*T_m_* at 343 °C) can be melt-processed below 400 °C due to a sufficiently low viscosity (flowability) [29]. Fine fiber nonwovens for hot gas filtration applications and membrane reinforcements were successfully produced [23,30]. Depending on the process parameters, average fiber diameters were obtained between 1.5 µm to 5.0 µm, which is within the common range of meltblown media made from other polymers. A limiting factor for industrial scaling for PEEK is the high material price. A further high-temperature polymer suitable for the meltblow process is PPS. Due to its structure containing aromatic rings linked by sulfur atoms, the polymer can be used up to 200 °C [31], but can already be processed at 300 °C. The polymer price of PPS is roughly one-fifth the price of PEEK. For meltblown nonwovens, PPS is already established and has been continuously developed since around 2013 [32]. Median fiber diameters range between 0.8 µm and 5.0 µm. However, compared to PEEK, a lower acid resistance and reduced strength must be accepted in addition to the reduction in application temperature (*T_g_* of PPS lies between 83 °C and 90 °C) [32].

The mechanical and thermal behavior of a thermoplastic polymer is not only governed by its chemical composition but also by its molecular structure, morphology, and processing history. In semicrystalline polymers, the crystalline phase and its morphology contribute to the material’s overall mechanical response, while the molecular orientation and crystallinity can influence properties such as stiffness, strength, and ductility [32,33]. Associated to this, *T_g_* is attributed to changes in the molecular chain mobility in amorphous regions, whereas melting and crystallization behavior is further based on the organization and affected by the thermal history of the polymer. Therefore, these thermal transitions provide useful context for interpreting material performance, but do not, on their own, explain the behavior of a processed polymer structure [34]. Applied on PEEK, PPS, and PESU, the role of the morphology differs between these materials in their structure–property relationship mechanisms as PEEK and PPS are semicrystalline while PESU is amorphous [35,36]. For PEEK, processing conditions and post annealing have been reported to affect crystallinity and mechanical properties, demonstrating that thermal history can influence performance without changing the polymer formulation [35]. For PPS, post-treatment like annealing can promote secondary crystallization and increase the storage modulus, while the selected heat-treatment conditions can alter the balance between strength and ductility [37]. For PESU, its amorphous character and resulting thermal behavior provide an alternative case in which the crystalline morphology is not the basis for interpreting the material’s response [38]. In fibrous materials, such as nonwovens, the polymer-level structure (primary, secondary and tertiary structure) and thermal history interact with the architecture of the web. Fiber morphology and orientation as well as the quality and distribution of binding points through fiber–fiber bonding or rather fiber-fiber entanglement can also influence the mechanical strength, modulus, and deformation characteristics [39,40] of a web structure. Thus, differences in the mechanical behavior of analyzed nonwovens may reflect both the intrinsic structure–property relationships of the respective polymer itself and the resulting fiber and web structure influenced by their respective processing parameters. The relative impact of these factors must be considered when relating thermal behavior to the tensile properties of the nonwoven samples [35,40,41].

Previous research on PAES targeting fibers, especially from PESU, has primarily focused on solution-based techniques and is rather limited to the laboratory scale. These include membrane-related hollow-fiber structures and applications by the dry–wet phase inversion spinning technique [42] and dry-jet–wet-spinning with the use of several modifications [43,44,45] or electrospun fibrous membranes [46,47]. These studies generally assess the membrane morphology and separation performance, rather than the tensile behavior and thermal response of a self-supporting nonwoven web [46]. Although PESU melt-spinning has also been reported, the respective study of Zhang et al. used a plasticizing agent, limiting its direct comparability with neat, unmodified PESU [48].

In this study, the meltblow processing of neat PAES is investigated. This polymer group contains a variety of amorphous thermoplastics and belongs to the high-temperature polymers. Economically interesting is that the polymer price is currently in the range of PPS, even though PAES show significantly higher glass transition temperatures (≥200 °C) than PPS and even PEEK. Typical processing temperatures lie between 200 °C and 320 °C for PSU [49,50] and 320 °C to 390 °C [51] for PESU and thus lower than required to process PEEK.

In this study, all three types of PAES used industrially (PSU (polysulfone), PPSU (polyphenylene sulfone) and PESU (polyethersulfone)) were screened for their suitability for the meltblow process. The processing trials were conducted on a technical-scale meltblow line targeting the lower limit of deposable/windable grammage (≤10 g m^−2^) in order to achieve thin fabric layers of ≤30 μm. The produced nonwoven PAES webs were characterized (fiber diameter distribution, thickness, air permeability and mechanical properties) and benchmarked against PEEK and PPS meltblown nonwovens produced on the same line. Further structure analysis using WAXS (Wide Angle X-Ray Scattering) and thermal analysis (DSC) were conducted. In terms of sustainability objectives, the funding project is closely linked to the identification of potential candidates for the replacement of PFAS-related fluoropolymers. In addition, significant energy savings through lower processing temperatures is a further ecological and economic goal. A unique feature of meltblown nonwovens is that they can be produced with adjustable pore sizes and surface characteristics, which increases their application potential if successfully implemented [52].

## 2. Materials and Methods

### 2.1. Materials

**Figure 1:**
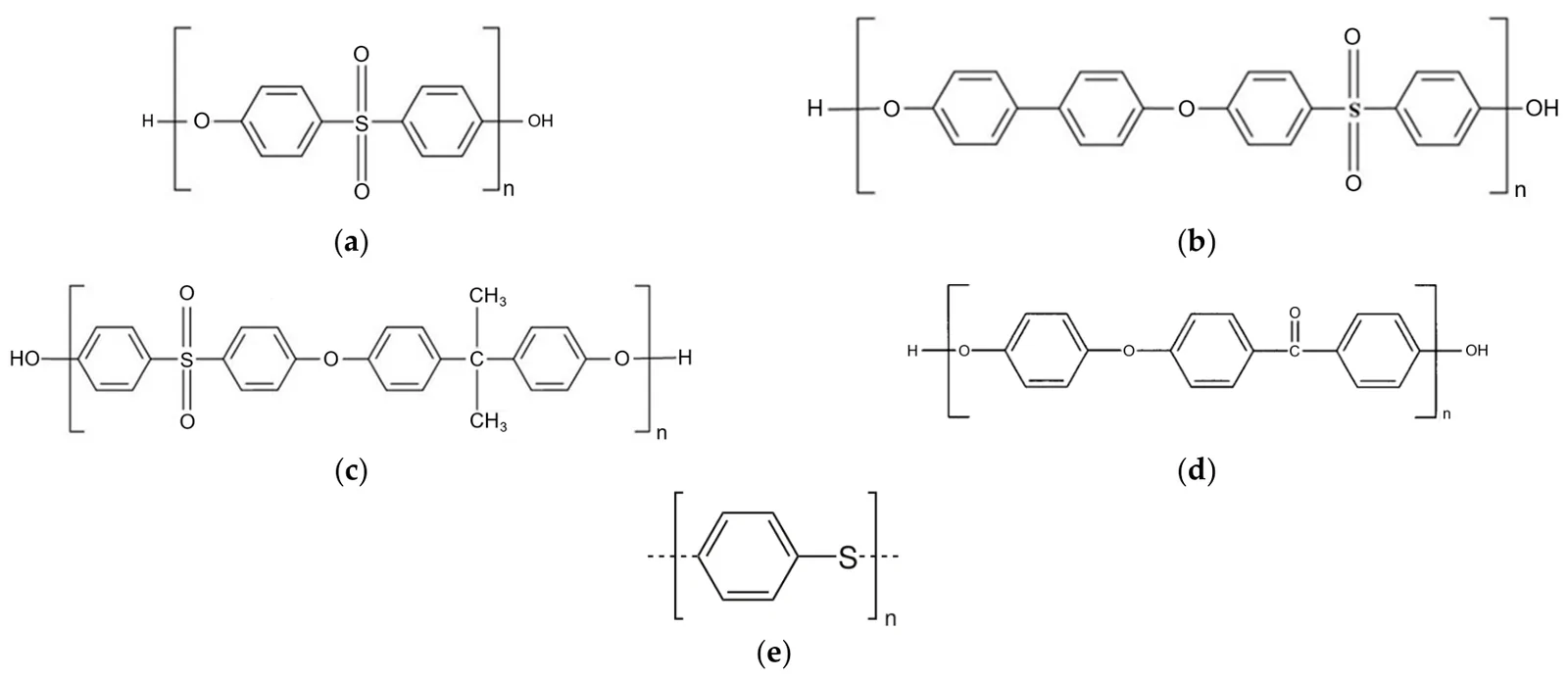
Chemical structures of the investigated polymer types: (**a**) polyethersulfone (PESU), (**b**) polyphenylsulfone (PPSU), (**c**) polysulfone (PSU), (**d**) polyetheretherketone (PEEK), and (**e**) polyphenylensulfid (PPS).

Polyarylethersulfones were supplied by BASF SE (Ludwigshafen, Germany). Different “Ultrasone*^®^*” types (PSU, PPSU, PESU) and grades were investigated regarding their processability. PEEK (Victrex 90G) and PPS (Fortron 0203P6) were used as reference materials. The chemical structures of the investigated polymers are shown in Figure 1.

All materials investigated in this study are summarized in Table 1.

### 2.2. Polymer Characterization

#### 2.2.1. Differential Scanning Calorimetry (DSC)

A Q2000 differential scanning calorimeter (TA Instruments Inc., New Castle, DE, USA) was used to determine the glass transition temperature and to detect potential recrystallization and melting peaks. Where applicable, *T_g_*, melt enthalpy *ΔH_m_* and *T_m_* were determined from the heat flow–temperature curves. The sample mass ranged from 2 mg to 10 mg. The heating and cooling rate was 10 K min^−1^. The samples were analyzed in a temperature range from room temperature (~25 °C) to 400 °C. Two heating phases and one cooling phase were carried out. Granules of the raw materials as well as fiber material from produced nonwovens were analyzed respectively.

#### 2.2.2. Determination of Moisture Content

Coulometric Karl-Fischer-Titration method was carried out to determine residual moisture content of the polymer granules after pre-drying. A “899 Coulometer” and a “885 Compact Oven SC” (both: Deutsche METROHM GmbH & Co., KG, Filderstadt, Germany) were used at 140 °C. The measured moisture content was below 0.015 wt.% for all polymers.

#### 2.2.3. Rheological Characterization

A “Physica MCR 501” rheometer (Anton Paar Group AG, Graz, Austria) equipped with a parallel-plate geometry (diameter 25 mm) was used to run shear rheological experiments in the temperature sweep and time sweep modes. The gap was adjusted to 1.0 mm and temperature ramps (heat rate: 2 K min^−1^, strain: 10%, angular frequency: 10 rad s^−1^, nitrogen atmosphere with flow rate of 50 mL min^−1^) performed while automatically adjusting the gap to maintain a constant normal force over the measurement. The strain amplitude was adjusted to the linear viscoelastic regime by strain sweep tests at a constant angular frequency of 10 rad s^−1^. Measurements in the time sweep mode (strain: 10%, angular frequency: 10 rad s^−1^) were conducted at selected process temperatures to evaluate the thermal stability of the materials.

#### 2.2.4. Wide Angle X-Ray Scattering (WAXS)

X-Ray Diffraction (XRD) measurements were conducted on a D/Max Rapid II diffractometer (Rigaku Corp, Akishima, Japan) using monochromatic Cu Kα radiation (*λ* = 0.15406 nm; *U_acc_* = 40 kV; *I_acc_* = 30 mA) and an image plate detector. A scanning rate of 0.2° min^−1^ and a step size of 0.1° were used. The measurement time was 1 h for all investigated samples. The obtained two-dimensional diffraction patterns were converted to the corresponding diffractograms using 2-theta (2*θ*)-intensity conversion by the software 2DP Ver. 2.1.6 (2018) (Rigaku Corp, Akishima, Japan). The diffraction patterns were analyzed using the PDXL 2.0 software.

The tests were executed using the produced nonwovens in order to detect the influence of the production process on the crystal formation. Therefore, the samples were prepared by arranging nonwoven sheets parallel to the sample holder carrier.

For the determination of diffraction peak positions and crystalline fraction, the diffraction patterns were fitted with pseudo-Voigt profiles (Equation (1)) in the range of 2*θ* = 10–40° to differ between the integrated intensities amorphous reflexes *I_a_* and of crystalline reflexes *I_c_*

(1) *I_a_* = *I*(2*θ*) = *I_hkl_* ⋅ [*f* ⋅ L(2*θ* − 2*θ*_0_) + ((1 − *f*) ⋅ G(2*θ* − 2*θ*_0_)]

with *f* being the Lorentz fraction (0 < *f* < 1), L(2*θ* − 2*θ*_0_), and G(2*θ* − 2*θ*_0_) being suitable Lorentz and Gaussian functions. The degree of crystallinity *χ_c_* was calculated according to Equation (2):

(2) $$\chi_{c}=\frac{\sum I_{c}}{\sum \left(I_{c}{+I}_{a}\right)}$$

*with*: *I_c_* = *I_total_* − *I_a_*

### 2.3. Nonwoven Processing

#### 2.3.1. Meltblow Set-Up

Meltblow processing trials were carried out on a technical scale meltblow line of 500 mm working width. The system was briefly described in earlier publications [63]. The spinneret was a 561-hole Exxon-type spinneret (28.4 holes per inch (*hpi*)) with capillary diameters (D) of 0.3 mm with a length to diameter ratio (*L/D*) of 8. The critical spinneret pressure is 50 bar; therefore, a safety limit was set to 45 bar during all experiments. The maximum polymer throughput was therefore limited by the melt viscosity of the respective polymer.

**Figure 2:**
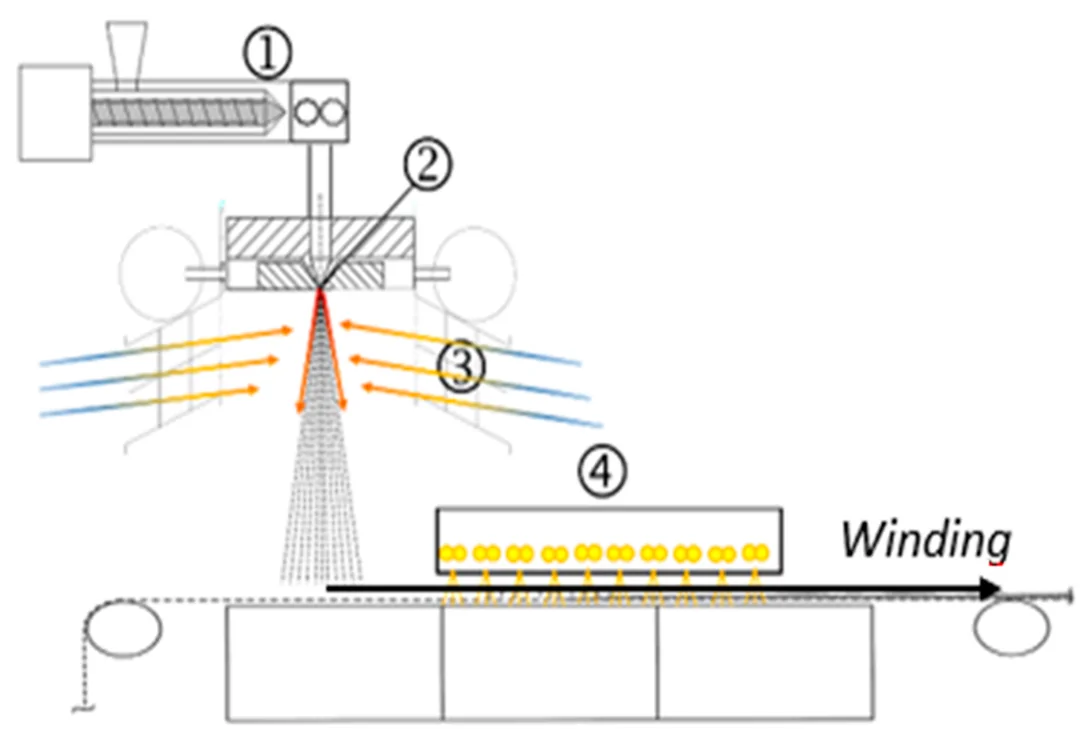
Scheme of the meltblow process adapted with heating elements for the elimination of thermal shrinkage: (1) extrusion system, (2) spinneret, (3) secondary air heaters, and (4) infrared heater; arrows: secondary air (from surrounding (blue: cold, red: hot).

To reduce the thermal shrinkage, commonly observed in meltblown high temperature polymers under reduced crystallization time [26], two passive secondary air heaters (SAH) of G. Maier Elektrotechnik GmbH (Reutlingen, Germany; dimensions: 700 × 120 × 100 mm) were installed below the spinneret on both sides parallel to the spinneret (see “3” in Figure 2).

This so-called “NABLO” technology heats up the secondary air [64], which passively gets entrained into the adjoining heat segments of each heater (volume: 350 m^3^, speed: 1.5 m s^−1^; air flowing through 36 U shaped VA tubular radiators of 8.5 mm diameter mounted on offset with 5.5 mm intermediate space), increasing the temperatures in the spinning room between the spinneret and the conveyor belt (max. by 150 K) as a result [65]. To the sides (CD), the open space between the spinneret and the SAHs was shielded by sheet-metal side panels to prevent cold secondary air from entering between those heaters. In addition, a post-treatment with an infrared heater “MX 500/810” by Heraeus Noblelight GmbH (Kleinostheim, Germany; (“4” in Figure 2)) was installed downstream of the fiber laydown point to provide thermal post-treatment of the deposited nonwoven. The infrared heater has an effective emitting area of 500 × 600 mm^2^ by five integrated shortwave infrared emitter twin-tubes (arranged across the conveyor belt with each (adjustable) 1.0 kW maximum heat output), which is positioned at a distance of 150 mm over the belt and 200 mm after the fiber deposition point.

#### 2.3.2. Meltblow Trials

Meltblow trials were conducted to produce nonwovens from the polymers listed in Table 1, which were identified as suitable for meltblow processing based on their rheological behavior (Section 2.2.3). Processing temperature and polymer throughput were systematically varied to find a stable processing window for each material. The melt temperature was adjusted by varying the temperature settings of the extruder zones, die zones and the spinning head based on the results of the rheological characterization (see Section 2.2.3) of the respective material with the objective of reaching a complex viscosity below approximately 100 Pa s in the Newtonian regime at low shear rates.

The meltblow line was operated at a polymer throughput ranging from 0.029 to 0.083 g ho^−1^ min^−1^ depending on the corresponding die pressure with the respective polymer (in total 1.410 to 2.91 kg h^−1^) and a conveyor belt speed of 3.95 to 9.75 m min^−1^ in order to produce fabrics with a target base weight of 10 g m^−^^2^.

Additional process parameters were adjusted individually for each polymer to ensure stable fiber formation at the spinneret and a uniform, shot-free fiber laydown on the conveyor belt. These include the following:

- Die-to-collector-distance (*DCD*): 300–500 mm;
- Temperature of secondary air heaters: max. 200 °C;
- Emitting power of the infrared heaters: max. 16.7 kW m^−2^;
- Volume flow of process air: 220–325 Nm^3^ h^−1^.

#### 2.3.3. Fabric Calendering

Calendering was performed using a customized laboratory thermobond calender by WUMAG TEXROLL GmbH & Co., KG (Krefeld, Germany) equipped with smooth rolls and a nominal working width of 600 mm. The calender is suited for processing high-temperature polymers and enables contact temperatures of up to 360 °C. Processing speeds between 0.5 and 15 mmin^−1^ and line loads from 10 to 60 kN can be applied. The roll gap is defined mechanically by an adjustable stop. Those parameters were varied according to the investigated polymer and experimental design. A siliconized paper by BRANOpac (Lich, Germany) with a thickness of 45 µm has been used as protective layers on the top and bottom, due to the fragile texture of the PESU meltblown fabrics. The production speed of all trials displayed in Table 2 was 1.0 m/min.

The calendering temperature was kept below the glass transition temperature (*T_g_*) of PESU to limit chain mobility and prevent structural deformation (or loss), which is contrary to semicrystalline thermoplastics. Here, this limit is generally determined by *T_m_*. respectively. 

### 2.4. Testing of the Physical and Mechanical Properties of Nonwovens

#### 2.4.1. Scanning Electron Microscopy (SEM)

The fiber diameter distribution was determined using scanning electron microscopy (SEM). Round samples were sputtered in an argon plasma (40 s under a vacuum of 0.1 mbar, with a distance of 35 mm, a current of 33 mA, and a voltage of 280 V) using a “Union SCD 040” by Balzers Union Ltd. (Balzers, Liechtenstein) with a gold–palladium layer of 10 nm to 15 nm. SEM micrographs were taken with a magnification of ×1000 using a “TM-1000 tabletop electron microscope” by Hitachi High-Tech Corporation (Tokyo, Japan) with an accelerating voltage of 15 kV in the “charge-up reduction mode”. The magnification was chosen to capture approximately 40 single fibers per image. Contrast and brightness were adjusted to gain an image of straight monochromic fibers in front of a dark monochrome background. To perform a software-assisted analysis of the fiber diameter distribution in the images, the beta software “MAVIfiber2d” of Fraunhofer ITWM (Kaiserslautern, Germany) was used [66]. First, the images were smoothed by an algorithm and binarized by the software before statistical analysis was performed over each fiber pixel without segmentation into individual fibers [67,68]. After merging the output of three images per sample, the mean and median fiber diameter as well as the standard deviation and interquartile range were determined.

#### 2.4.2. Fabric Area Base Weight

The area base weight was determined referring to DIN EN ISO29073-1. The sampling size was adjusted to 100 cm^2^ (10 cm × 10 cm). At each measuring position along machine direction (MD), three samples were taken in cross direction (CD) and averaged to consider homogeneity scattering across the production width.

#### 2.4.3. Fabric Thickness

The fabric thickness was measured according to DIN EN ISO 9073-2 using a test head of 25 cm^2^ by Frank-PTI GmbH (Birkenau, Germany) and a test force of 5 cN cm^−2^. Eight measurements were conducted diagonally along the sample, determining a median value for thickness (*δ*).

#### 2.4.4. Air Permeability

Air permeability was measured in accordance with EN ISO 9237:1995-12 with a sample size of 20 cm^2^ and a differential pressure of 200 Pa. The equipment used was an FX3300 by TEXTEST AG (Schwerzenbach, Switzerland).

#### 2.4.5. Tensile Testing

To evaluate the mechanical performance, tensile tests were executed using an “Instron UPM 4301” by Instron GmbH (Darmstadt, Germany) with a 5 kN measuring head with pneumatic clamps of 100 mm clamping length. The tensile strength (*σ_m_*), elongation at peak force (*ε_m_*), elongation at break (*ε_B_*), and the elastic modulus (*E*) as secant modulus were characterized, each in MD and CD respectively. For each nonwoven sample, five specimens with a width of 15 mm were tested. The sample thickness was determined individually as described in Section 2.4.3 and the median of five measurements was used for the calculation of the stress from the recorded force. The test speed was set to 100 mm min^−1^. The tenacity was calculated using the sample dimension, the fabric thickness, and the measured peak force. The median and the standard deviation of all measurements’ properties were used to compare the nonwoven characteristics.

#### 2.4.6. Thermal Stability

The heat shrinkage behavior of the fabrics was characterized according to the “drying oven method” (ISO 11501:1995 and GB/T 12027–2004) [69]. Rectangular samples (300 mm × 100 mm in MD) were taken from the left, in the middle and the right side of a nonwoven sample. The specimens were placed free-hanging in an oven at 200 °C for 30 min.

## 3. Results and Discussion

### 3.1. Processability Analysis of Polyarylethersulfones

**Figure 3:**
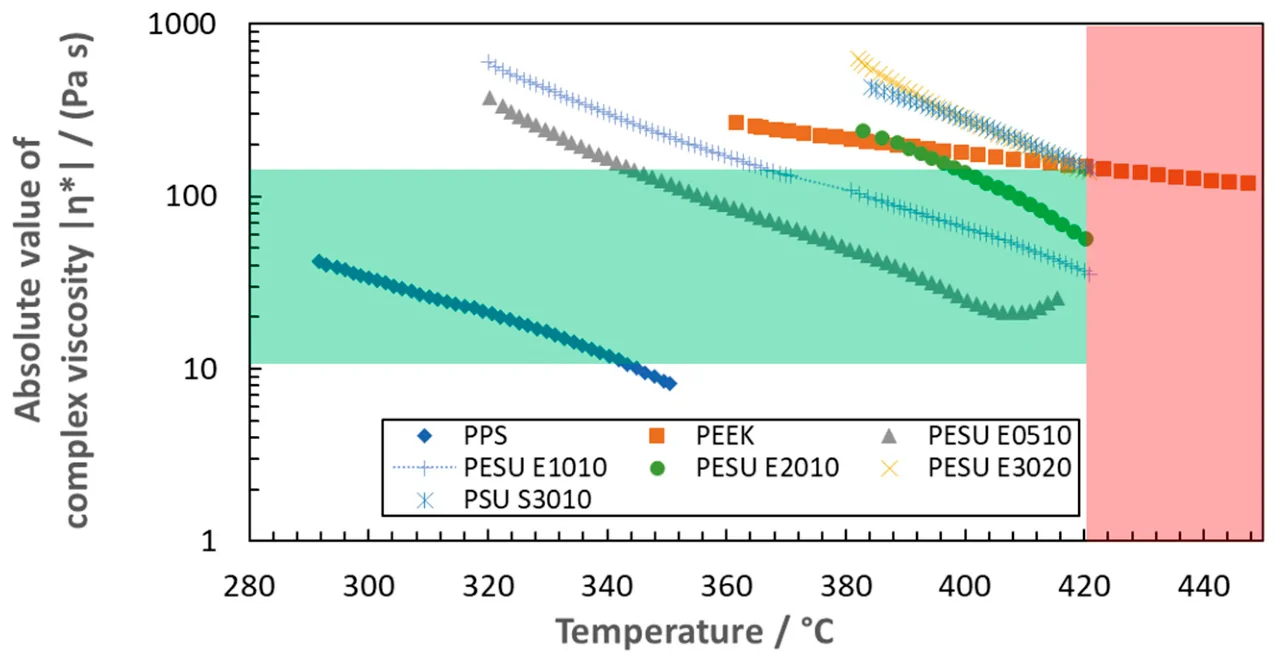
Temperature-sweeps of the complex shear viscosity at low shear (*ω* = 10 rad s^−1^) of selected polyarylethersulfone materials; green: target window for meltblow processing; red: exceeding max. machine temperatures.

**Figure A1:**
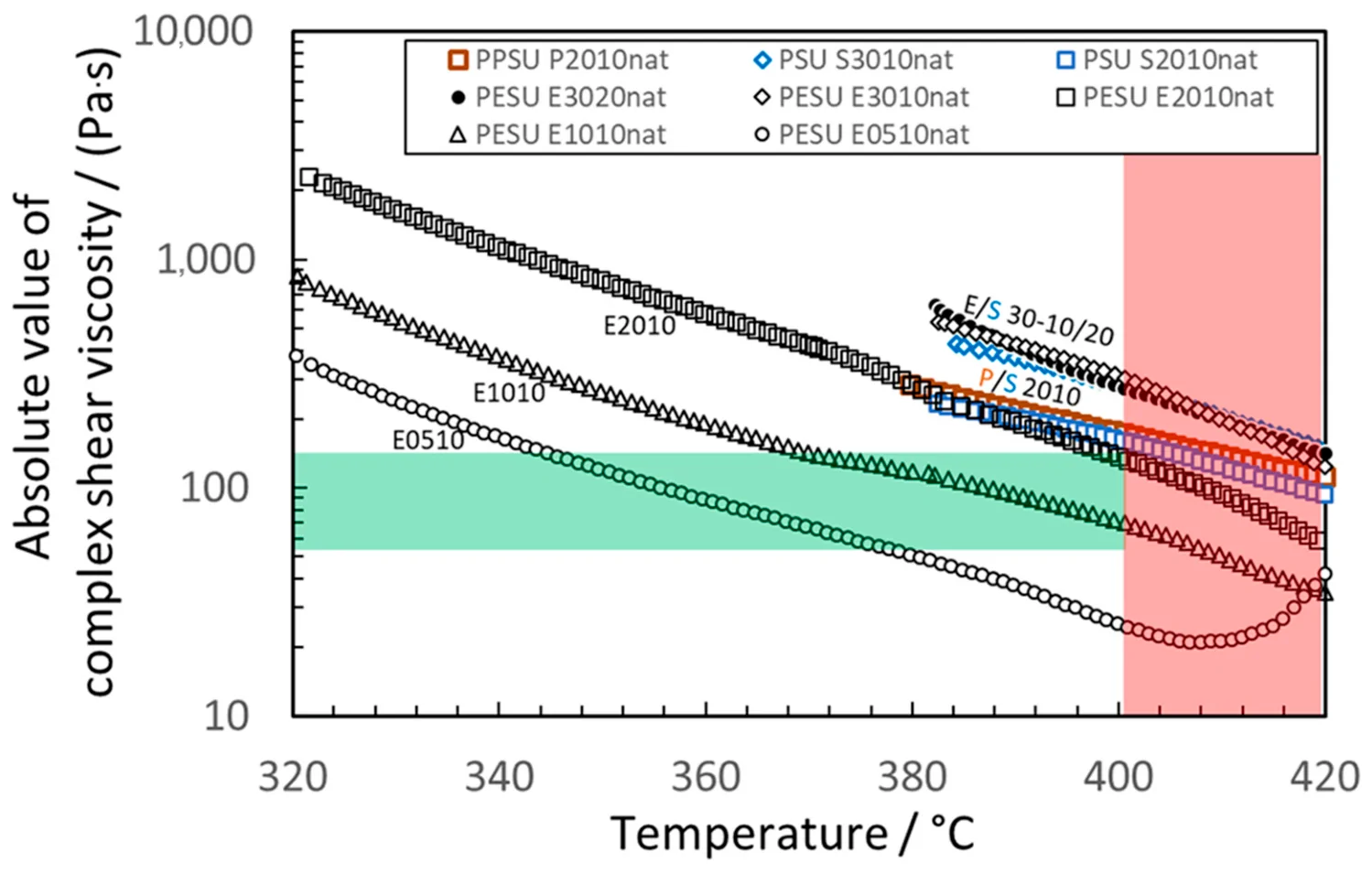
Temperature sweeps of the complex shear viscosity at low shear (*ω* = 10 rad s^−1^) of all screened polyarylethersulfone materials; green: target window for meltblow processing, red: outside possible machine temperatures.

Figure 3 shows the temperature-dependent complex shear viscosity of the selected polyarylethersulfones. PAES grades showing a higher viscosity range were excluded from this figure to reduce complexity. A complete plot comparing all screened samples is given in the Appendix (Appendix A.1, Figure A1).

One of the key material requirements for successful meltblow processing is that the molten polymer exhibits sufficient flowability when passing the spinneret in order to ensure a non-critical pressure level. Further, the extruding filaments need to be taken up by the acting force of the primary air stream to be drawn easily. Thus, the correlating material property, the viscosity, has to be very low compared to other polymer processing methods including filament spinning. However, high shear rates with more than 1000 s^−1^ can occur by flowing through the die capillaries, which is the first crucial step for fiber formation. Capillary or slit-die rheometry would provide a more representative viscosity range within the power-law regime, since such high shear rates are not accessible with rotational shear-rheometers. However, this employed method provides a second important quantity: the storage modulus, which is a measure of the elastic energy stored in the viscoelastic melt. Previous studies [23,47,70] have shown that the complex start-viscosity of time-sweeps at low shear rates (Newtonian regime) as well as the complex viscosity of temperature-sweeps are more suitable values for a valid assessment of the processability than the melt flow index [23]. Based on these studies, an upper complex viscosity limit in the range of 100 Pas to 150 Pa s has been identified for different polymers to unlock them for the meltblow process.

This rheological requirement is fulfilled by two of the investigated PESU grades, Ultrason*^®^* E0510nat and E1010nat. The viscosity of a third PESU grade, E2010nat, falls below the specified limit only at the highest investigated temperature and is excluded from further consideration. This is also the case for all other PESU grades, the two PSU grades and the PPSU sample, which all remain insufficiently flowable over the measured temperature range. This observation is noteworthy because PSU shows a lower glass transition temperature and is expected to soften at lower temperatures compared with PPSU and PESU. However, within the investigated range of polymer grades, PSU reveals a comparatively weak temperature dependence of its rheological properties.

**Figure 4:**
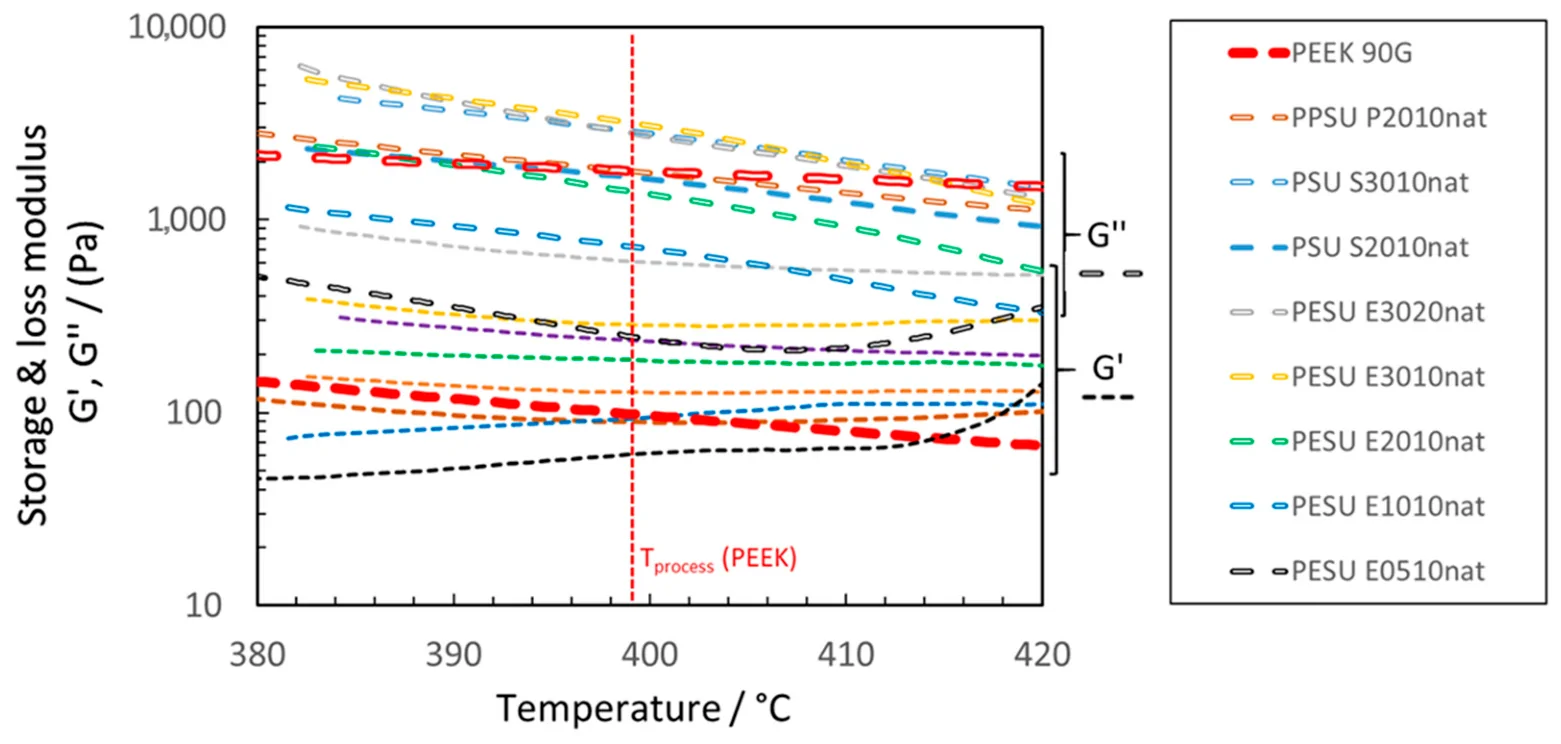
Storage and loss modulus from shear-rheological temperature-sweep of the selected polyarylethersulfone materials in reference to PEEK; vertical red dashed line: process temperature of PEEK in the meltblow process.

For the PAES, the corresponding storage and loss moduli as a function of the temperature are provided in Figure 4 supplemented by the moduli of PEEK as a reference high-temperature polymer for meltblow processing.

The complex, the storage and loss moduli of PEEK and PPS at their respective processing temperature viscosity at low shear rates (Newtonian regime) are further given in Table 3.

As a first indication of their processability, it becomes apparent that the loss and storage moduli of all three PAES types are of the same order of magnitude as those of the PEEK reference material, which has already been demonstrated to be suitable for stable meltblow processing. In contrast to PEEK, the storage moduli of the PAES grades increase slightly with increasing temperature. This may be an indication for structural changes, even though the effect is not particularly pronounced. PESU E0510nat, which clearly shows the lowest viscosity level, exhibits a more pronounced increase in both *G*′ and *G*″ at temperatures exceeding 410 °C. This temperature range is irrelevant for processing, but underlines the previous thesis of structural build-up like cross-linking, branching, or thermal degradation. However, for all investigated PAES grades, *G*″ remains higher than *G*′ over the entire investigated temperature range, indicating predominantly fluid-like behavior without a crossover of *G*″ and *G*′. Additionally, it should be noted that PPS also shows an increase in *G*′ between 270 °C and 350 °C as was reported in an earlier study [23].

**Figure 5:**
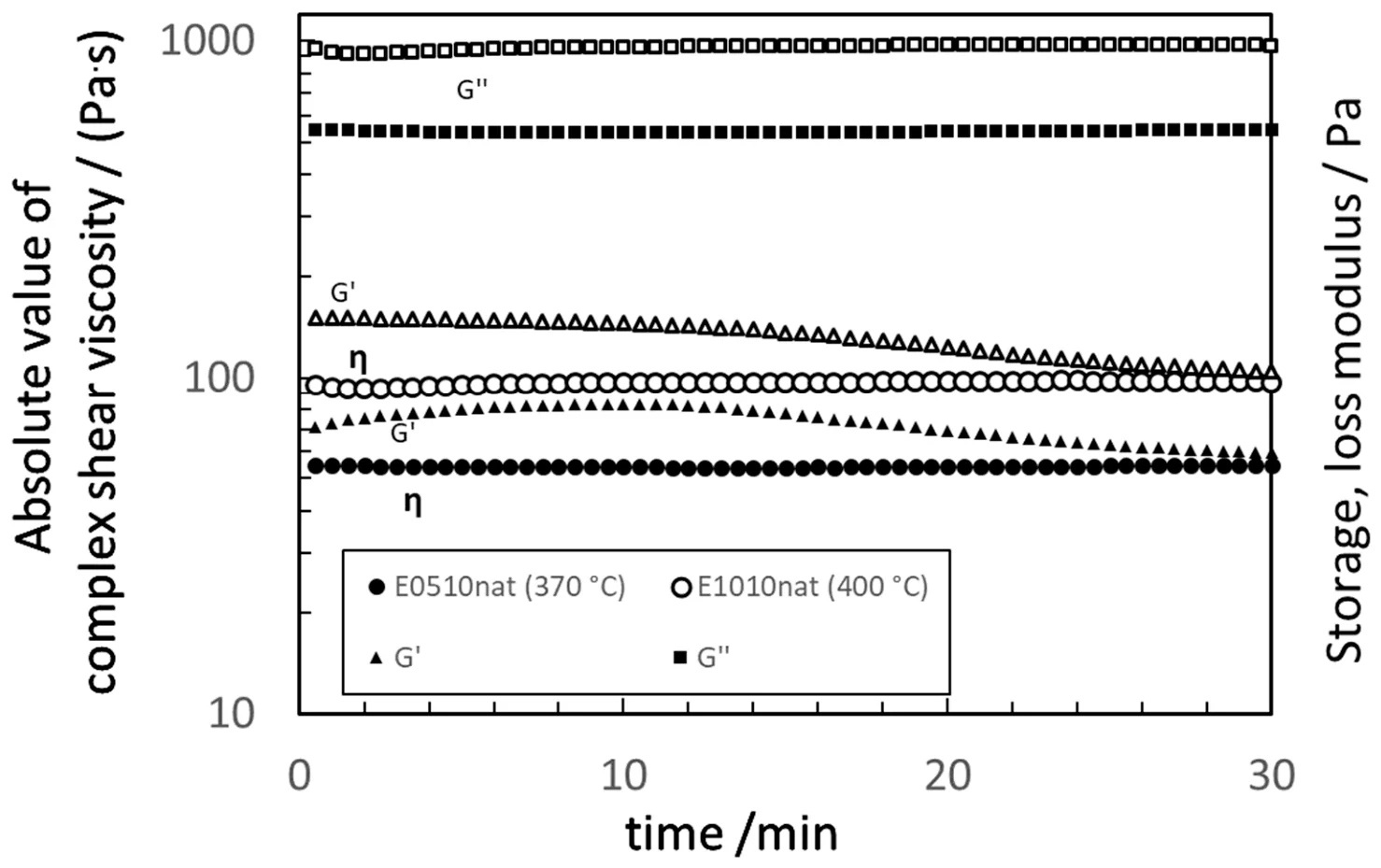
Time-sweeps of the complex shear viscosity (η), storage (*G*′) and loss modulus (*G*″) at low shear (ω = 10 rad s^−1^) of the two selected PESU grades.

The temperature-sweeps, performed to evaluate the thermal stability of the grades selected for further consideration (PESU E0510nat and E1010nat), are shown in Figure 5.

Despite the slight initial increase in *G*′ observed for E0510nat, both materials exhibit behavior similar to that of PEEK and PPS [23], and exhibit stable rheological behavior over the residence time in the rheometer, which is required for a stable extrusion process without degradation effects.

### 3.2. Meltblow Processing

Both PESU types identified in Section 3.1 were successfully processed using the meltblow technology. Table 4 shows the process parameters for different settings including the reference materials PPS and PEEK and the two selected PESU grades with the goal to achieve the finest fiber diameters possible while maintaining a certain productivity.

For PPS, the air flow rate was varied to investigate its influence on the resulting fiber diameters. In a further step, the temperatures of the melt and process air were moderately increased, combined by a significant reduction in the throughput. This adjustment was possible due to the lower melting range of this polymer, the greater distance to the degradation range and, in particular, the very low viscosity, resulting in low process pressures in this temperature range and higher flexibility. It should be noted that the per-hole-throughput of 0.034 g ho^−1^ min^−1^ corresponding with a total throughput of below 2.3 kg h^−1^ m^−1^ lies on the lower end of relevance compared to 0.072 g ho^−1^ min^−1^ (~4.9 kg h^−1^ m^−1^) and 0.089 g ho^−1^ min^−1^ (~6.0 kg h^−1^ m^−1^) of PESU 0510. Slight deviations in the throughputs of PEEK and PESU E1010 refer to the different densities of the materials and the volume-controlled processing conditions. However, the lower throughput of PEEK and PESU 1010 compared to PESU E0510 displays the limitation of productivity due to the high die pressure. This indeed relates to the comparably high viscosities (close to 150 Pa s at 395 °C for PEEK and 100 Pa s of PESU 1010 at 400 °C). A further consequence of the higher viscosity and melt elasticity (reflected by storage modulus) is that the process air had to be maximized to the amount deliverable by the compressor at its maximum operation point (1.35 bar) at the respective process temperature. This subsequently results in a very narrow process window together with the comparatively harsh processing conditions. The low viscosity of PESU 0510, which is closest to that of PPS, allowed lower process temperatures to be used and also allowed the melt and air temperature to be varied in order to examine their respective effects on the fiber formation. The resulting differences in fiber diameters or mechanical properties are not solely attributable to polymer chemistry directly. However, the necessary adjustments of processing parameters for the different polymers are indeed related to the polymer class and type as well as to their structures, molar mass and correlated properties. This is especially the case for the extrusion and air temperatures and also for the throughput limitations, which, in turn, necessitate adjustments to the belt speed to achieve the targeted grammage.

### 3.3. Nonwoven Characterization

**Figure 6:**
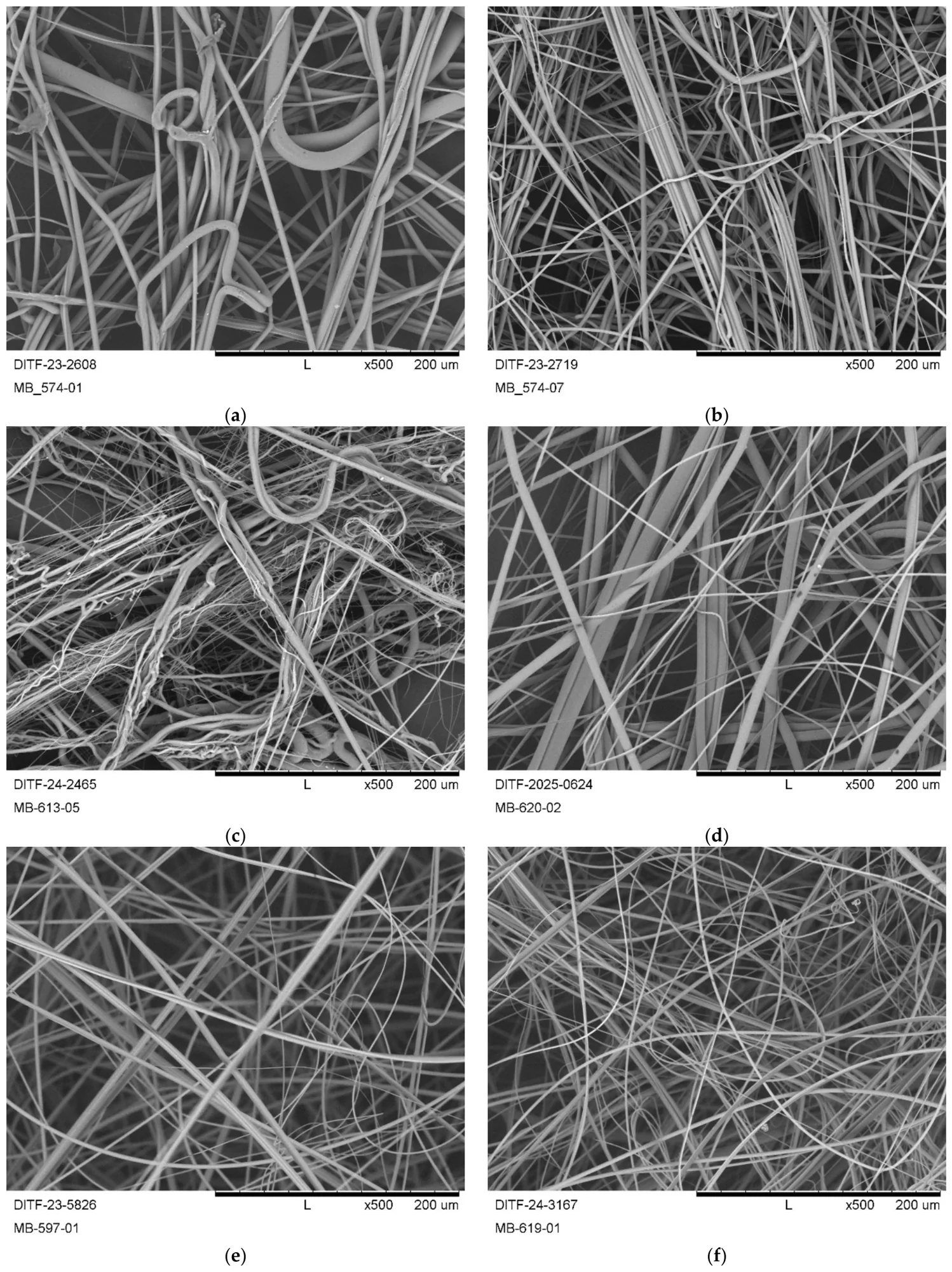

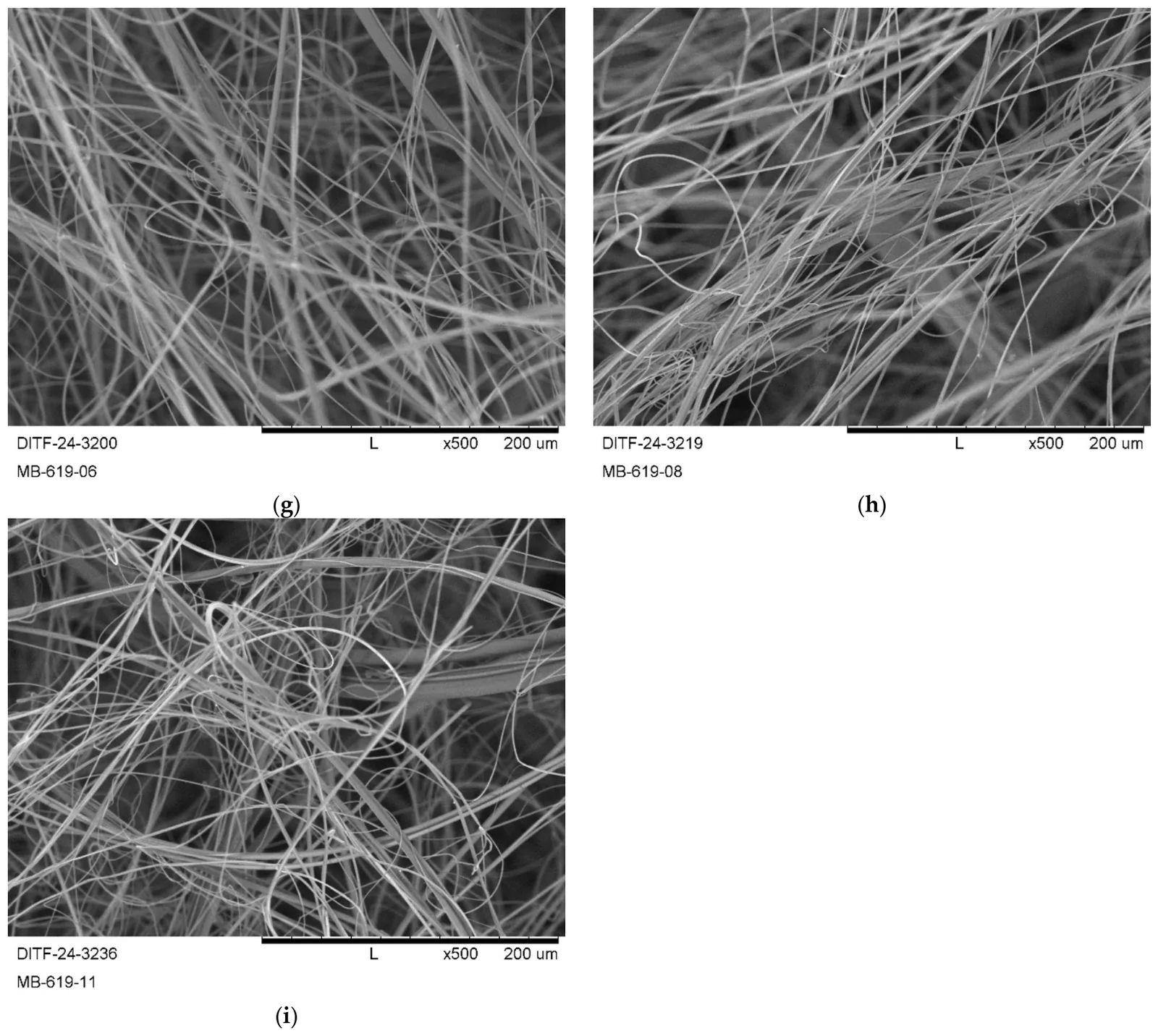
Microscope images (SEM) of produced nonwoven samples in 500-time-magnification: (**a**) PPS-01, (**b**) PPS-02, (**c**) PPS-03, (**d**) PEEK, (**e**) PESU-1010, (**f**) PESU-0510-01, (**g**) PESU-0510-02, (**h**) PESU-0510-03, and (**i**) PESU-0510-04.

**Figure A2:**
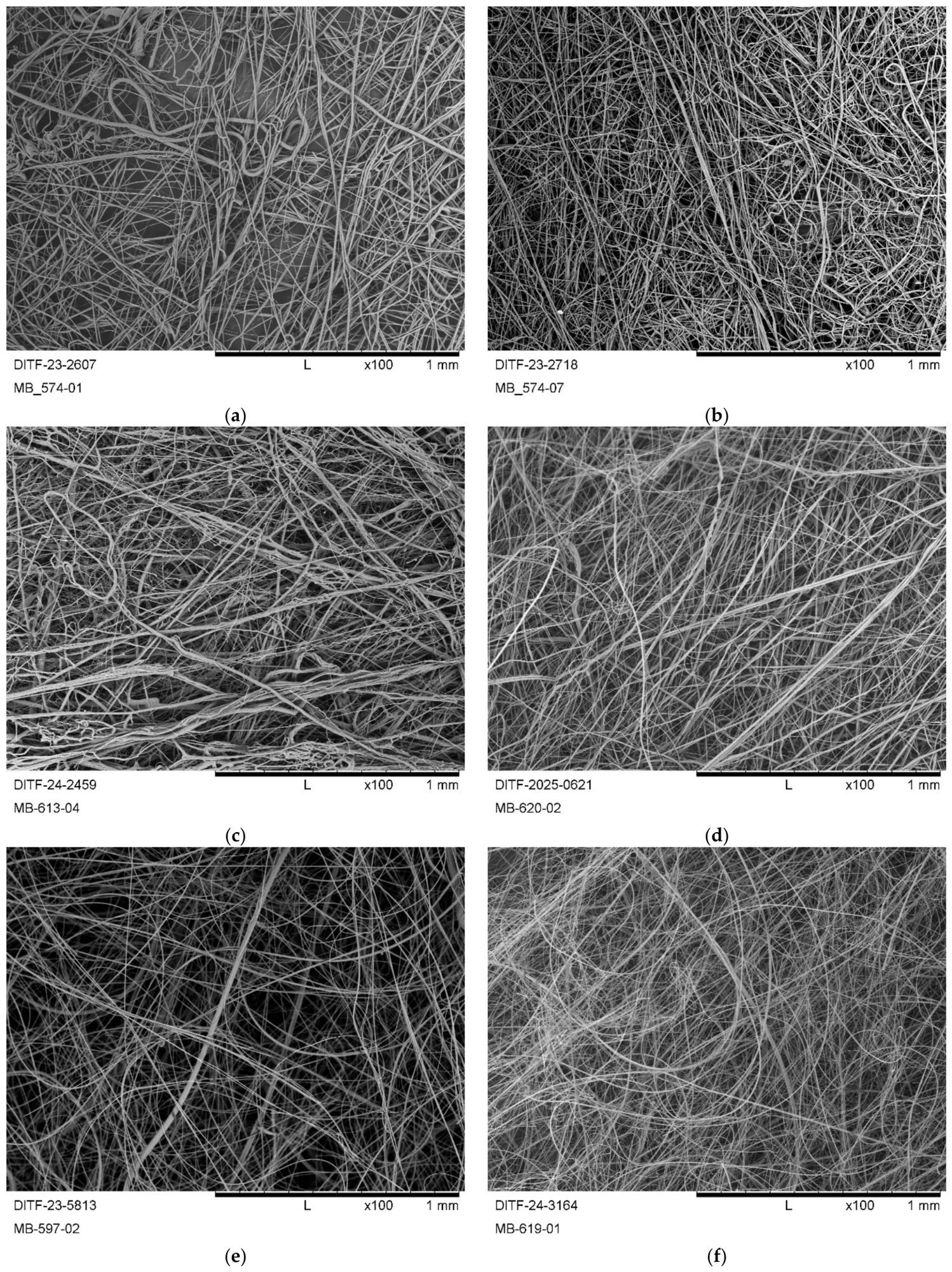

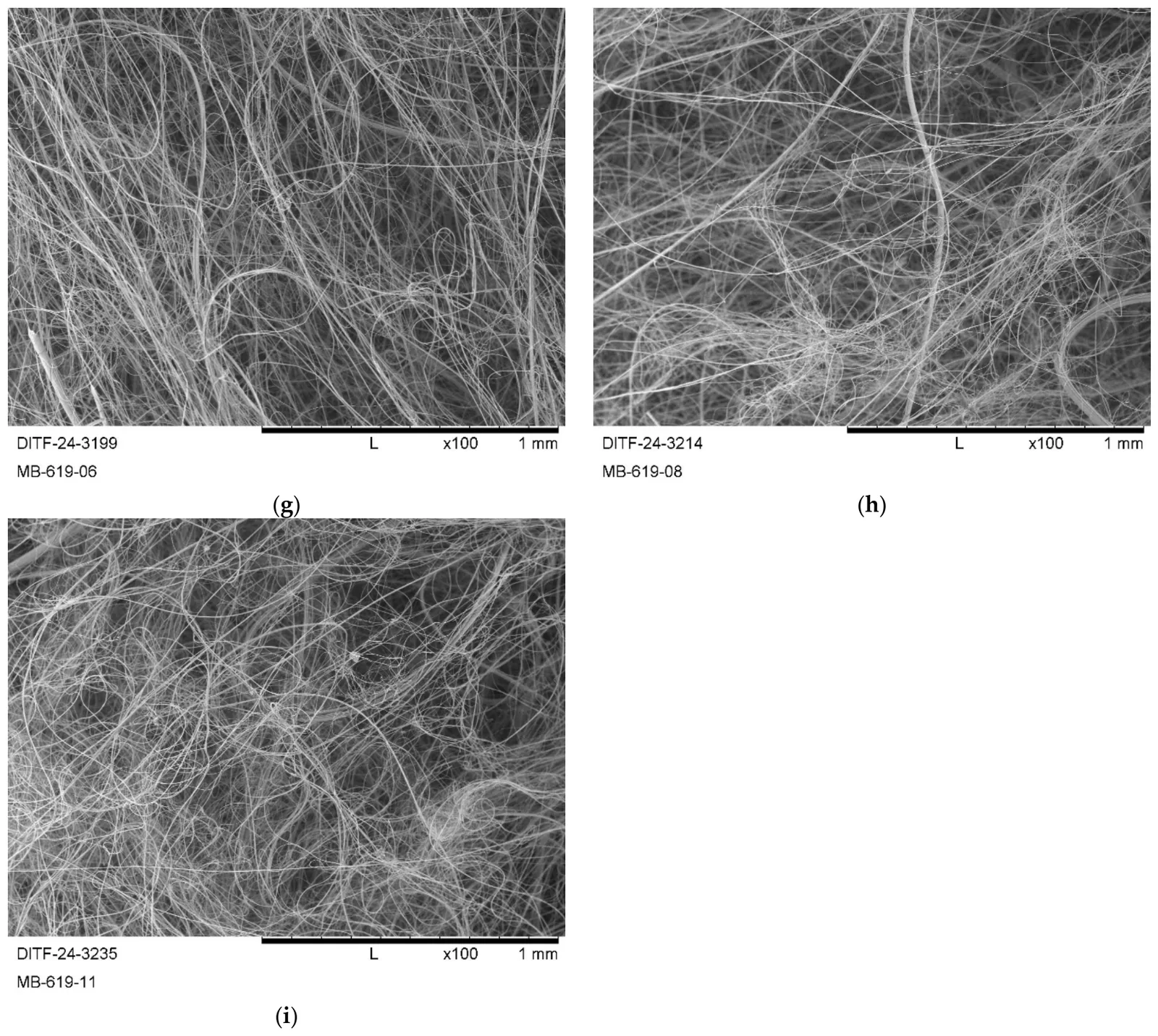
Microscope images (SEM) of produced nonwoven samples in 100-times magnification: (**a**) PPS-01, (**b**) PPS-02, (**c**) PPS-03, (**d**) PEEK, (**e**) PESU-1010, (**f**) PESU-0510-01, (**g**) PESU-0510-02, (**h**) PESU-0510-03, and (**i**) PESU-0510-04.

Figure 6 shows micrographs by SEM in the 500-times magnification of each process setting of the different materials. Further micrographs in 100-times magnification are shown in the Appendix (Appendix A.2, Figure A2).

Qualitatively, the fibers from PESU (Figure 6e–i), particularly those in the in-focus regions, appear brighter, almost transparent in contrast to the other materials as an indicator for their amorphous character.

**Figure 7:**
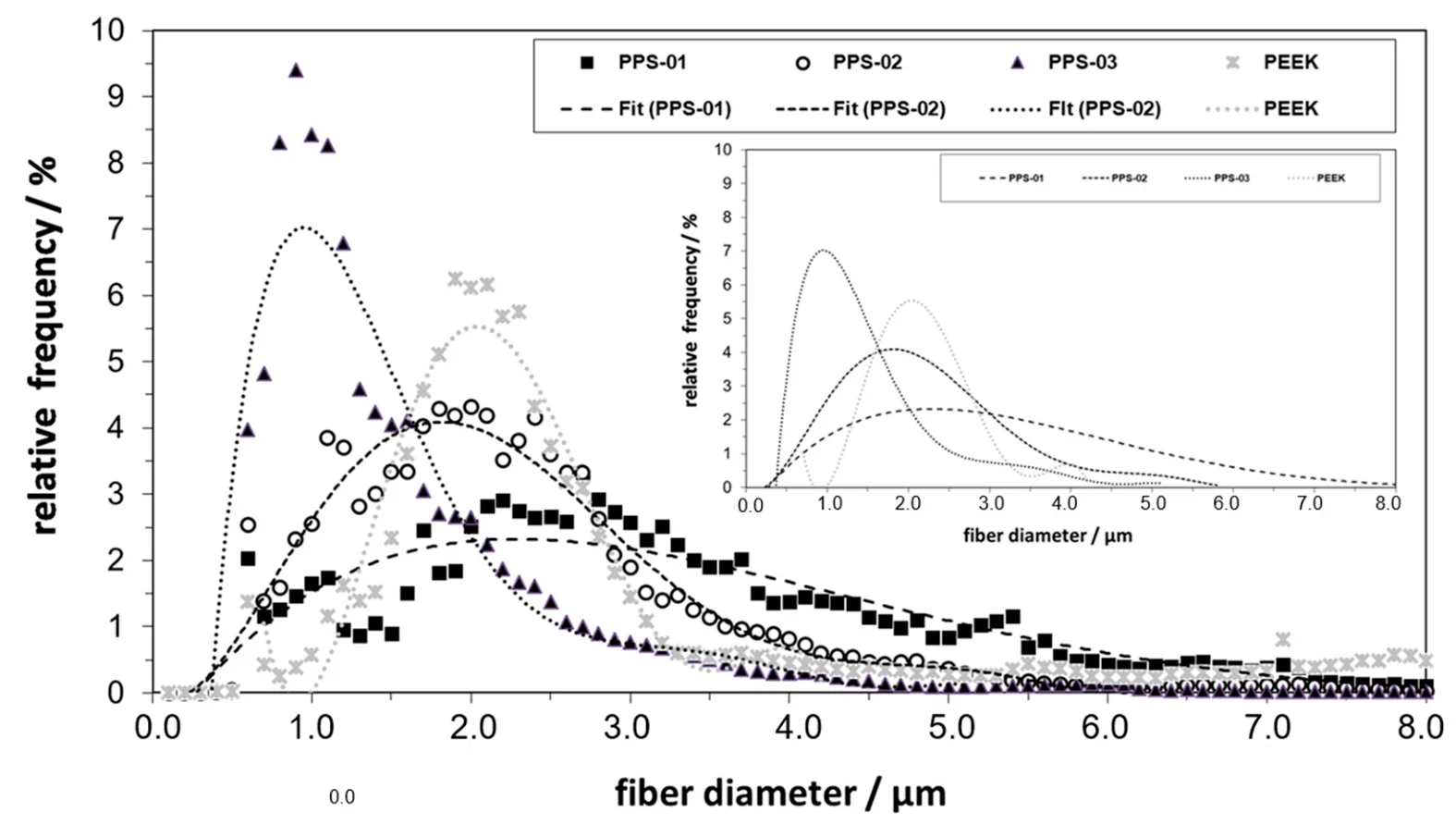
Fiber diameter distributions of the produced nonwovens of the reference materials PPS and PEEK; fits: polynomials, also separately displayed in small windows.

**Figure 8:**
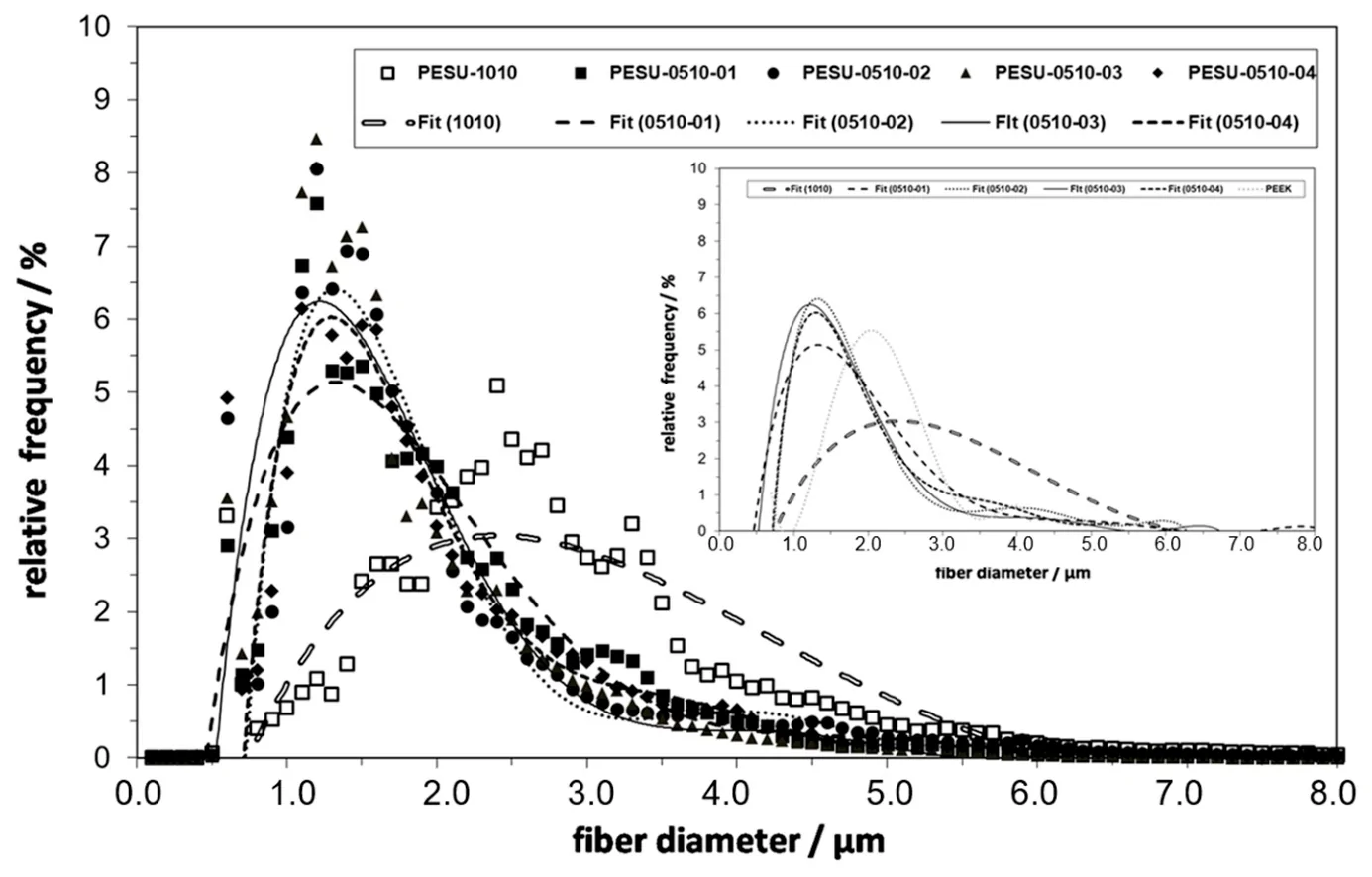
Fiber diameter distributions of the meltblown PESU nonwovens, grey dots: distribution of PEEK (reference); fits: polynoms, also separately displayed in small windows.

**Figure 9:**
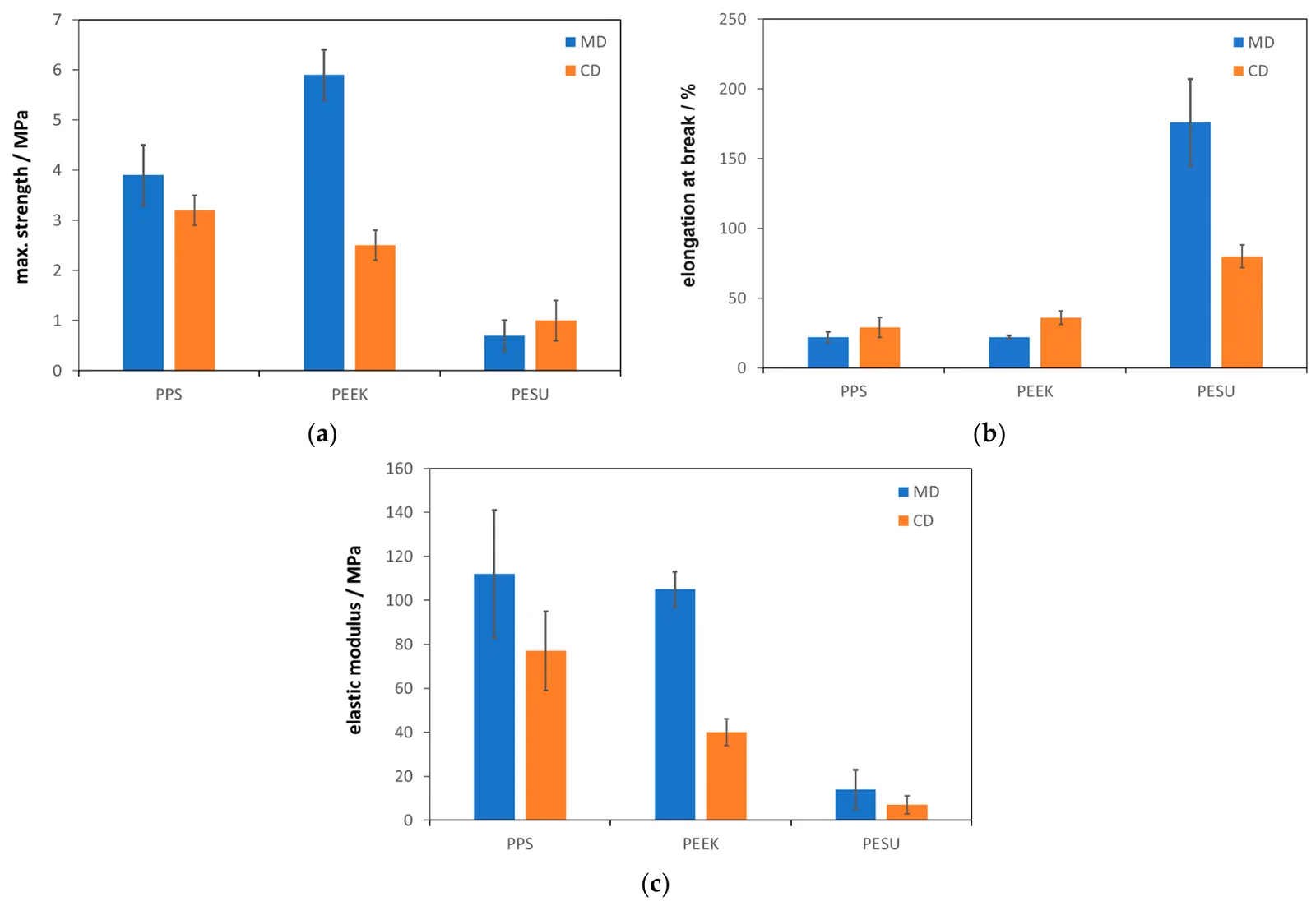
Average mechanical nonwoven properties of the different polymer materials: (**a**) max. strength, (**b**) elongation to break, and (**c**) elastic modulus.

Quantitatively, the fiber diameter distributions resulting from the SEM images are compared in Figure 7 for PPS and PEEK and in Figure 8 for the meltblown PESU and Figure 9 for centrifugal-spun PESU nonwovens.

Both the qualitative micrographs and, more clearly, the quantitative evaluation of the fiber diameters reveal the influence of the process air increase (Figure 6a vs. Figure 6b) and the reduced polymer throughput (Figure 6c). The distribution develops from a diffuse, broad value scattering (PPS-01) to an increasingly narrower distribution. The distribution of the higher viscous PEEK is also expressed as equally distinct, but shifted to higher fiber diameters in average (Figure 6d), consistent with its higher melt viscosity and the resulting reduced attenuation of the melt by the process air.

Moreover, a clear evolution to finer and more distinct distributed fiber diameters can be observed from PESU 1010 (Figure 6e) and over an increasing melt and air temperature for PESU 0510 (Figure 6f–i). While the PESU 1010 fabric shows a diffuse broad distribution peak, the peaks of PESU 0510 are shifted to lower fiber diameters but showing no significant differences from one another as the temperature increases. A statistical hypothesis test (Pearson’s chi-square test of homogeneity) is provided in Appendix A.3. For a more detailed insight, the median and mean average values of the fiber diameter distributions are given in Table 5 together with further nonwoven characteristics (area weight, fabric thickness and air permeability) tested on the samples.

As indicated before, PESU 1010 shows a broad fiber diameter scattering and high average values, comparable to PEEK. Both also were characterized before, with a comparable shear-unaffected high viscosity ≥ 100 Pa s at their process temperature. But, PPS also shows even coarser fiber diameter averages when processed with a lower air amount. However, the reason for the more distinct distribution of PEEK vs. PESU 1010 may be related to differences in their shear-thinning behavior. The lowest average fiber diameters were achieved with PPS, but, as previously discussed, under a very low productivity (per-hole-throughputs) of the meltblow line. Indeed, the progressing temperature increase on PESU 0510 resulted in a continuous lowering of the average fiber diameter, from 1.63 µm at 358 °C of melt and 390 °C of air to 1.39 µm at 386 °C (melt)/410 °C (air). The latter conditions approach the upper temperature range of conventional meltblow equipment. In practice, the energy aspects and component lifetime must certainly also be weighed up, potentially accepting slightly higher fiber diameters (10–18%).

However, median or average fiber diameters < 1.0 µm, which can be achieved with other, low-viscosity polymers in the meltblow process [71], were not achieved under the conditions investigated here. The reason for this is the high process temperature, which is necessary to reduce the viscosity and achieve the necessary flowability. Nevertheless, the viscosity level of PEEK grades with a low molar mass is still comparatively high, which limits the access to higher polymer throughputs (productivity).

The nonwoven specifications, such as the base weight as the production target, are equally low within all samples. Due to the low areal coverage of the fibers, the air permeability of the low grammage structures is also quite high throughout all samples. This counts especially for the air permeability of PESU 1010 with an open structure of rather coarse fibers.

However, apart from the fiber analyses and nonwoven properties already shown, a significant difference was found in the web formation of the fabrics produced and in the mechanical stability of the materials as a direct consequence. Therefore, without the use of secondary air heaters and the infrared post-heaters, all four HT-polymers formed fluffy fabrics with almost no mechanical strength. For PEEK and PPS, this is primarily due to the substantial initial temperature drop (up to 70 K) that occurs directly after the fibers leave the meltblow die [23] and relatively slow crystallization kinetics compared to polymers like PP with quite linear molecular structures. If the highly oriented polymer chains are not stabilized by crystallization, the relaxation of the molecular orientation upon reheating above T_g_ can contribute to fiber shrinkage and dimensional instability. This can result in a high thermal shrinkage and poor mechanical stability of the nonwoven fabrics. A related effect has previously been reported for PET, for which a fiber crystallinity of approximately 35% was identified as a threshold for suppressing this behavior [26].

These issues were successfully eliminated for the two semi-crystalline thermoplastic materials PPS and PEEK by precisely controlling the material temperature throughout the process all the way from the spinneret up to the fabric winding, utilizing the described SLE and IR treatment (see Section 2.3.1). This treatment provided additional time at elevated temperatures (above T_g_) for crystallization and structural stabilization [26,72]. The resulting nonwoven fabrics showed a significantly denser and thinner deposition structure and a more “paper-like” haptic.

To quantify these “haptic impressions”, the mechanical performance of the nonwoven made from the different materials is compared in Figure 9 through the results of tensile testing (see Section 2.4.5). The displayed columns for each polymer are mean values of the discussed processing settings.

As discussed above, PESU fabrics show inferior mechanical properties. In particular, the combination of low strength and high elongation illustrates that the deposited fibers are only loosely entangled and can easily slide off each other. Without significant force absorption, the nonwoven structure can be dissolved by slipping and pull-out during deformation. Due to the comparably high *T_g_*, the fibers reach the freezing point quickly after exiting the die, resulting in a fabric where there are almost no physical (thermally merged) binding points among the PESU fibers. However, the tensile data in MD or CD alone do not establish that the comparatively high glass-transition temperature (*T_g_*) causes insufficient fiber–fiber bonding. More generally, the mechanical response of nonwovens depends not only on the properties of the constituent fibers, but also on fiber orientation, web structure, and the quality and distribution of bonding points [40,73,74]. The resulting MD/CD ratio of the PESU nonwovens provides additional evidence consistent with this interpretation, although it does not independently confirm the proposed mechanism.

**Figure 10:**
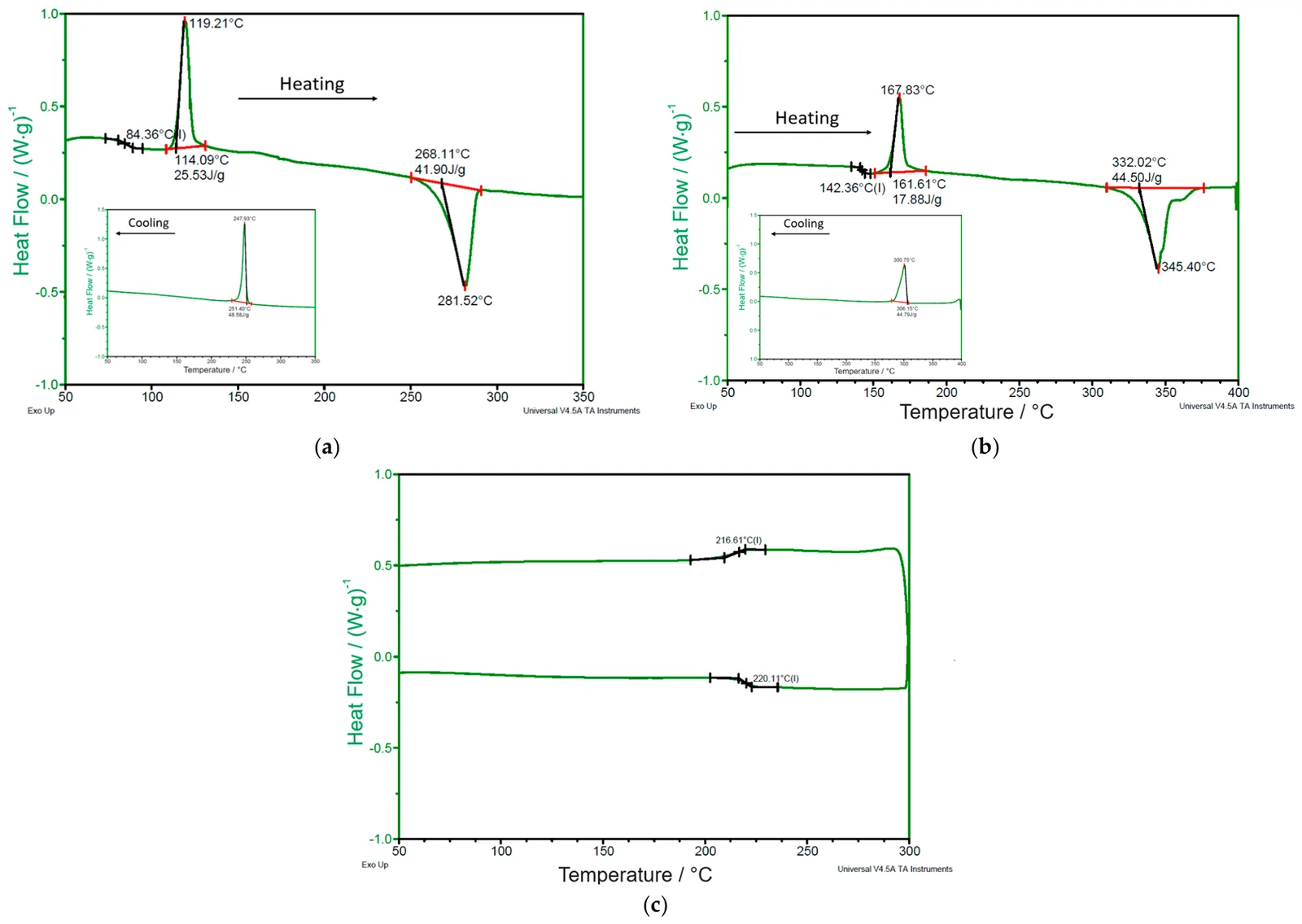
DSC thermograms of selected nonwoven fabrics of (**a**) PPS (-03), (**b**) PEEK and (**c**) PESU (-02).

PEEK shows superior strength against PPS in machine direction (MD). However, in the presented setting, PPS shows nearly a balance of equal MD to CD properties with a higher strength and modulus in the cross direction than PEEK. As a possible reason, this can be attributed to the turbulent process air acting less effective in stretching the higher-viscosity PEEK, but keeping the fibers more strongly aligned in MD. This also agrees with the coarser mean fiber diameters, but, by itself, it does not necessarily refer to the differences in fiber morphology [40,75]. The direction-dependent (anisotropic) mechanical behavior in nonwovens can be influenced by the fiber orientation, bonding, and web structure [39,40]. The observed mechanical anisotropy is therefore consistent with the differences in processing behavior and potentially stronger MD fiber alignment, but this hypothesis remains unconfirmed because fiber orientation was not measured directly. A balanced MD-to-CD ratio of the mechanical properties is often a desirable target attribute for isotopically loaded applications alongside the absolute values in each direction. Summarizing, the results are consistent with the influence of the molecular structure. As the error bars represent samples produced under different processing conditions, it becomes evident that the relative contributions of the polymer type and processing conditions cannot be separated from this comparison alone [39,40,76]. For semicrystalline thermoplasts such as PEEK, the crystallization behavior and mechanical properties can depend on the molecular structure and thermal processing history; studies of molded PEEK/PAEK materials have reported changes in the tensile strength and modulus with crystallinity and thermal treatment [77]. These findings provide general structure–property context but cannot be transferred quantitatively to the present fibrous nonwoven system. To reveal the degree of crystallization induced by the meltblow process, the DSC thermograms of selected meltblown fabrics of each material are shown in Figure 10.

**Figure 11:**
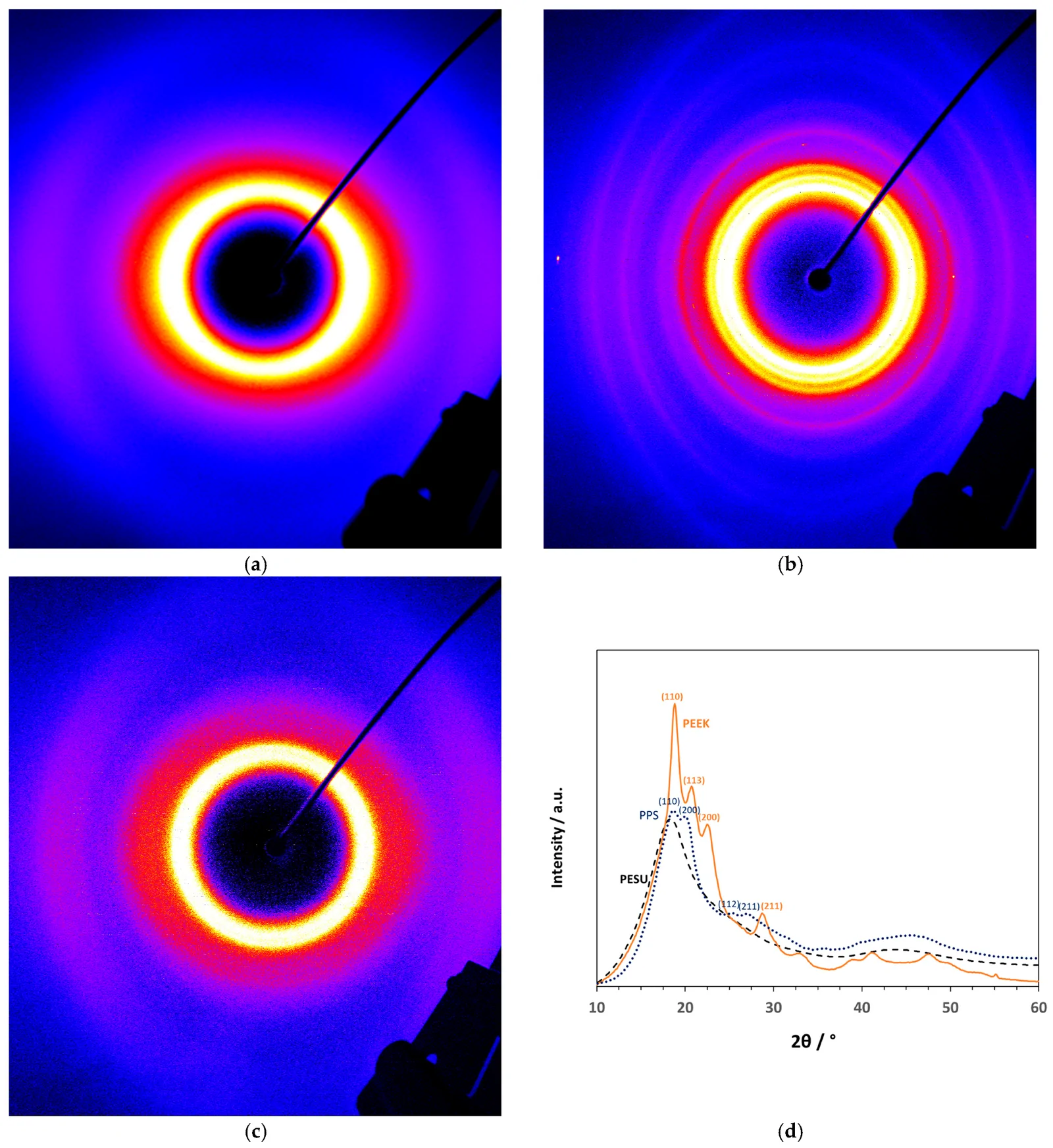
WAXS diffraction images and corresponding diffractograms/patterns of selected meltblown nonwovens based processed from high-temperature polymers: (**a**) PESU (05), (**b**) PEEK, (**c**) PPS (04) and (**d**) diffractograms (black dashes: PESU (05); orange line: PEEK and blue dots: PPS (04); *the selected samples shown are representative for the respective polymer type).*

For both PPS and PEEK, distinct melting and crystallization peaks are observed. The residual crystallization peaks indicate that the fibers did not crystallize completely during processing, likely because of the short times available for crystallization during fiber formation and cooling. This indicates that the full crystallization potential of the polymers is not yet reached by applying the heat treatment during and after the meltblow process. Based on the standard melt enthalpies (*ΔH_m,0_*) of both materials from the literature (77.5 J g^−1^ for PPS [78] and 130 J g^−1^ for PEEK [79]), the crystallinity in the fibers can be calculated by subtracting the crystallization peak enthalpy from the melting enthalpy and normalizing the result by the standard melting enthalpy. Thus, crystallinity values (formed during fiber cooldown) of around 21% result for PPS-03 and ~20% for PEEK in relation to their respective potential. PESU, as expected, shows only the glass transition without recrystallisation and melting in the heating or recrystallization during the cooling run. This is confirmed by the structure analysis on the nonwoven materials by WAXS, shown in Figure 11.

While PESU, as an amorphous polymer, (expectedly) forms completely amorphous fibers, shown by the diffraction image (Figure 11a) and the corresponding pattern (Figure 11d), PEEK shows a discrete semi-crystalline structure (Figure 11b). Several crystalline reflections occurred at 2θ = 18.8°, 20.7°, 22.5°, 28.7°, 32.9°, 38.9°, 41.2°, 47,5°, 55.1° and an additional shoulder at 50.0°, agreeing with the literature [80,81,82,83]. Here, the first four mentioned reflexes assigned to the lattice planes ((111), (113), (200) and (211)) are also alternatively reported to refer to (213) [80,81] of the orthorhombic unit cell of the reported crystal structure of PEEK [82,83]. For PPS, which is also reported to crystallize in the orthorhombic structure [84,85], an almost amorphous XRD pattern was obtained. Similar diffuse diffraction patterns were observed in earlier works on partially and low oriented PEF-fibers, produced by melt spinning [86], but in combination with a high anisotropy of the parallel-aligned fibers, which cannot be seen by the random-oriented fibers in the nonwoven structure examined in this work. Moreover, the nonwoven/fiber structure generates a very pronounced amorphous halo that almost completely overlay the crystalline structure; the observed peaks are in accordance with the literature [84,85,87,88] and align with the characteristic *(hkl)*-planes of the orthorhombically unit cell at 2θ = 18.6° (110) and 2θ = 20.0° (200) [84,85]. Further reflexes at 25.4° and 27.1° are connected to the (211) and (122) plane [88]. Some reflexes are shifted compared to the literature values (18.6° vs. 18.8°/10.2°, 20.0° vs. 20.5°/20.8°, 25.4–25.7°, 27.1° vs. 27.8° [84,88]), which indicate changes in the lattice parameters of a material, which can be attributed to various factors such as stress, crystal size, temperature, or doping. A shift to lower 2*θ*-values corresponds to an increase in interplanar spacing (d-spacing) according to Bragg’s Law, indicating lattice expansion, microstrain, or unit cell distortion.

The degree of crystallinity in the PEEK and PPS nonwovens was determined from the diffractograms using “pseudo-Voigt profiles” over the range of 2θ = 10–40°, resulting in 39% each (39 ± 7% for PEEK and 39 ± 5% for PPS). Consequently, the crystallinity determined by WAXS exceeds that measured by DSC by approximately 18–19 percentage points. This systematic difference may arise from the distinct physical phenomena measured by the two techniques. WAXS detects the total instantaneous volume fraction of ordered domains in the solid state at room temperature, including contributions from the rigid amorphous phase (RAF). In contrast, DSC measures the enthalpy of fusion (*ΔH_m_*) during dynamic thermal melting, where the RAF does not undergo a distinct thermodynamic melting transition within the baseline integration limits. Nevertheless, in the DSC measurements, the degree of crystallinity was equal for both semi-crystalline polymers and is close to the required crystallization level of 25% stated by Rieger [26] to successfully suppress recrystallization by re-heating above *Tg*. The difference, observed between the crystallinities resulting from DSC and WAXS of at least 10–20%, can be due to over- and underestimation by the respective test, and is further neglected by considering the standard deviation of the WAXS results (5% & 7%), assuming a similar scattering in the DSC.

The X-RAY pattern of PESU only shows an amorphous halo. Unlike semicrystalline polymers, which develop crystalline regions during cooling, amorphous PESU does not undergo crystallization to provide additional crystalline reinforcement. Consequently, the mechanical integrity of the PESU nonwoven may need to be improved through post-treatment, for example, by thermobond calendering (see Section 2.3.3). The roll temperature was set to 200 °C and, thus, below *T_g_* (222 °C), which is different from semi-crystalline thermoplastic materials (PPS, PEEK), which can, or, rather need to be, calendered above *T_g_* as the critical temperature is *T_m_*.

**Figure 12:**
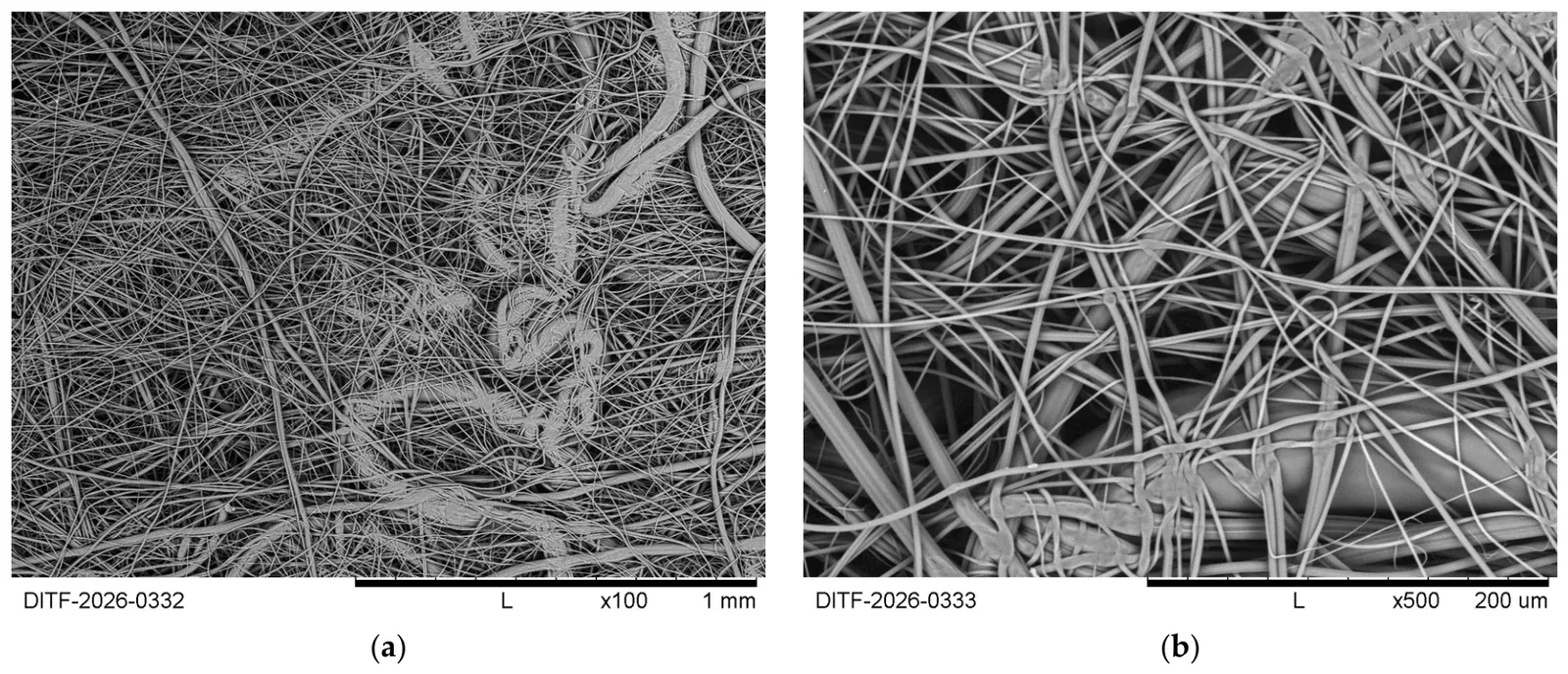
Microscope images (SEM) of a calendered nonwoven sample (PESU-KV2) in different magnifications: (**a**) 100-times, and (**b**) 500-times.

SEM micrographs of the calendered nonwoven in 100-times and 500-times magnification are given in Figure 12.

Already at 100-times magnification, the increased compactness of the fabric compared to Figure 6h (and Figure A2h) is clearly visible. This difference becomes even more apparent in 500-times magnification, showing the compressed contact points between the individual fibers due to mechanical and/or cohesive bonding. However, no thermal fusion could be observed in the SEM images. Compared to the displayed values of Table 4, a soft calendering of PESU-0510-02_KV2 already led to a decrease in thickness to 29 ± 5 µm at the cost of the air permeability, which was reduced to 2220 ± 295 L m^−^^2^ min^−1^. The overall strength of the fabric was increased and almost doubled in MD to 1.0 ± 0.25 MPa, reducing the corresponding elongation to 32 ± 9%. However, the measured maximum strength values remained below that of the reference materials (compare Figure 9a). The deviation in the presented mechanical values, particularly for PESU, is exceptionally high. However, this can be attributed to the loose, fluffy structure of the uncalendered samples, which complicate the sample extraction, preparation and general post-processing. Structural inhomogeneity in the initial web may also contribute to the variability observed after calendering. Given the small number of samples, these effects are more pronounced. However, the comparison to the reference materials is still significant and valid. For filtration and membrane support applications, air permeability and porosity are critical performance indicators.

Calendering also binds protruding fibers and loose surface loops into the nonwoven structure and thereby dramatically reduces the problematic hairiness, preventing fibers from catching and potentially damaging the fabric during handling or processing. As calendering was executed 20 to 30 K below *T_g_* of PESU, the effect is comparably low. However, higher calendering temperatures (close to and exceeding *T_g_*) led to instantaneous dimensional loss due to shrinkage and a complete loss of the nonwoven character of the samples. Based on the results of the structural analysis, the hot air shrinkage behavior of the selected materials at 200 °C (30 min exposure) is shown in the Table 6. The temperature setting of 200 °C was selected because it lies above the *T_g_* of the semicrystalline polymers PPS and PEEK but below the *T_g_* of PESU and, therefore, represents a relevant application temperature. The shrinkage in MD shows that the dimensions of all HT-polymers addressed in this study remain stable within this setting of 200 °C.

The measured shrinkage is limited and ranks PEEK < PESU < PPS in their uncalendered state. After thermobond calendering (KV), the shrinkage of PPS and PESU could successfully be further reduced to below 1%. For the semicrystalline PPS, the reduction in shrinkage may be attributed to both the relaxation of process-induced molecular orientation and residual stresses and the further increase in the crystalline segments. This is consistent with the nearly amorphous WAXS-halo (Figure 11) in the uncalendered state. For the amorphous PESU, the reduction in shrinkage after calendering below *T_g_* suggests at least a partial relaxation or rearrangement. However, the pronounced shrinkage observed upon heating above *T_g_* (qualitative finding) indicates that a significant fraction of the process-induced stresses remains within the fibers.

## 4. Conclusions

The meltblow processing of PESU was investigated and compared with the other high-temperature thermoplastics, PPS and PEEK.

While PEEK generally offers superior thermal resistance (long-term use temperature) and overall chemical resistance, the investigated PEEK nonwovens also exhibited the highest mechanical strength in the machine direction. However, PPS was found to generate smaller fiber diameters, which is also of interest to further application fields, like filtration. Furthermore, the processing of PEEK is limited by achievable polymer throughput (productivity) due to its high melt viscosity.

Suitable Ultrason*^®^* PESU grades were identified through rheological characterization, indicating their potential processability. Using the commercially available PESU grades with the lowest viscosity, average fiber diameters close to 1 µm, in the same range as those obtained for PPS, were achieved. However, the weak mechanical web strength of the generated PESU nonwovens makes thermomechanical post-treatment by calendering indispensable for further use. This could be related to the amorphous structure of the PESU polymer, which also remains present in the nonwoven fibers. As a result, the thermal control during the fiber formation and deposition seems to be less effective than it is known to be for semicrystalline polymers with a tendency for heat shrinkage due to slow and insufficient crystallization such as PET or PEEK.

Nevertheless, with the generated PESU meltblown, the processing of an amorphous high-temperature polymer to a nonwoven from melt was successfully demonstrated. In certain fields of application, the availability of PESU meltblown fabrics could provide an alternative to per- and polyfluorinated alkyl substances (PFAS) such as PTFE or PVDF, as very thin nonwoven layers could be produced in a productive single-line process (including calendering) and provide stability at a verified temperature of 200 °C.

A further point to be considered in the context of the direct-processing of PESU into nonwoven materials is that PESUs can also be processed using solution-based technologies, e.g., electrospinning and centrifugal spinning—unlike PPS or PEEK. Using these technologies, it is generally possible to form significantly smaller fiber diameters in the submicron or nanofiber range, which is especially interesting for applications in filtration and separation. Although the focus in this work was on melt-based fiber formation, the feasibility of the solvent-based fiber formation of PESU was demonstrated in this project as well and the results will be published separately.

Thus, in terms of sustainability goals, this study contributes to the identification of potential alternative materials to substitute PFAS-related fluoropolymers, such as PTFE or PVDF. For selected applications requiring elevated temperatures, the findings of this study could help to reduce the overall need for layers or coatings made from PFAS-related fluoropolymers, thereby achieve positive ecological, social and economic effects. Achieving energy savings by the processing of high-temperature-resistant, low-cost polymers to the finest fiber nonwovens at lower temperatures is also a worthwhile ecological and economic goal.

## Figures and Tables

**Table 1:** Material properties by supplier specification.

Polymer	Product	Grade	Supplier	*T_g_*, *T_m_* /°C ^a^	Density /(g cm^−3^) ^b^	MVR /(cm^3^ 10 min^−1^) ^c^	Data Sheet
PSU	Ultrason^®^ S3010nat	Unreinforced, higher-viscosity injection molding and extrusion grade, tougher and with improved chemical resistance.	BASF ^f^	187, -	1.23	55	[53]
	Ultrason^®^ S2010nat	Unreinforced, medium-viscosity standard injection molding grade.		187, -	1.23	95	[54]
PPSU	Ultrason^®^ P2010nat	Ultrason P 2010 is an unreinforced, medium-viscosity injection molding PPSU grade	BASF ^f^	220, -	1.29	-	[55]
PESU	Ultrason^®^ E3010nat	Unreinforced, higher-viscosity injection molding and extrusion grade, tougher and with improved chemical resistance,	BASF ^f^	228, -	1.37	34	[56]
	Ultrason^®^ E2020P	e.g., for membrane applications, coatings and resin modification, containing min. 50% of OH-endgroups		225,	-	Molar mass weight-average *M_w_*: 52,000 Da, *M_w_*/*M_n_*: 3 (SEC in DMAc, PMMA std.)	[57]
	Ultrason^®^ E2010nat	Unreinforced, medium-viscosity standard injection molding grade		225, -	1.37	70	[58]
	Ultrason^®^ E1010nat	Unreinforced, low-viscosity injection molding grade, flows very readily		222, -	1.37	150	[59]
	Ultrason^®^ E0510nat	Unreinforced, very-low-viscosity injection molding grade, good flowability		222, -	1.37	320	[60]
PPS	Fortron 0203P6	Easy flowing unfilled grade	Celanese ^d^	90, 280	1.35	-	[61]
PEEK	90G	Unreinforced, semi crystalline, depth filtered granules for injection molding, very easy flow	Victrex ^e^	143, 343	1.30	Viscosity: 90 Pa s at 400 °C (ISO 11443)	[62]

^a^ ISO 11357-1-3/ISO 3146; ^b^ ISO 1183-1; ^c^ ISO 1133-1; ^d^ Celanese Services Germany GmbH (Sulzbach Tauus, Germany); ^e^ Victrex plc (Lancashire, UK); ^f^ BASF SE (Ludwigshafen, Germany), *all listed PAES polymers of BASF belong to the Ultrason^®^ series*.

**Table 2:** Overview on the calandering processing parameters.

Sample ID	Temp. /°C	Gap /mm	Load /kN
PPS_KV1	150	0.3	60
PESU-0510-02_KV2	200	0.1	20
PESU-0510-02_KV3	210	0.1	10

**Table 3:** Rheological properties of PPS (Fortron 0203P6) and PEEK (90G) at their processing temperatures.

Polymer	Extrusion Temperature /°C	Absolute Value of Complex Shear Viscosity ^1^ /(Pa s)	Storage Modulus /Pa	Loss Modulus /Pa
PPS	297	36	5	360
PPS	315	24	11	235
PEEK	395	157	105	1857

^1^ determined in Newtonian and linear-viscoelastic regime.

**Table 4:** Overview of the meltblow processing parameters.

Sample	Extrusion Temp. /°C	Air Temp. /°C	Per-Hole Throughput /(g ho^−1^ min^−1^)	Die Pressure /bar	Belt Speed /(m min^−1^)	Air Flow /(Nm^3^ h^−1^)	DCD /mm	Secondary Air Temp. /°C	Infrared Intensity /(kW m^−2^)
PPS-01	300	305	0.072	10.1	8.0	220	300	100	11.7
PPS-02	300	315	0.072	9.5	6.0	275	300	100	11.7
PPS-03	315	330	0.029	5.5	4.5	220	500	100	5.3
PEEK	396	415 *	0.034	26.7	3.5	235 *	300	240	11.7
PESU 1010	400	415 *	0.069	27.7	8.0	285 *	250	140	11.7
PESU 0510-01	358	390	0.083	16.6	4.7	285 *	250	240	11.7
PESU 0510-02	368	395	0.083	13.9	4.7	285 *	250	240	11.7
PESU 0510-03	377	400	0.083	12.8	4.7	285 *	250	240	11.7
PESU 0510-04	386	410 *	0.083	7.9	4.7	285 *	250	240	11.7

* max applicable (deliverable) amount by compressor at max. working pressure (1.3 bar).

**Table 5:** Resulting fabric characteristics of the nonwovens.

Sample	Area Weight /(g m^−2^)	Median Fiber Diameter /µm	Mean Fiber Diameter /µm	Fabric Thickness /µm	Air Permeability /(L m^−^^2^ min^−1^)
PPS-01	11 ± 1	2.94	3.53	25 ± 2	3730 ± 145
PPS-02	10 ± 0.5	2.07	2.30	26 ± 2	2850 ± 475
PPS-03	9 ± 1	1.23	1.57	38 ± 4	3170 ± 1190
PEEK	10 ± 1	2.10	3.03	31 ± 6	2780 ± 590
PESU 1010	11 ± 1	2.57	3.25	± *	6450 ± 220
PESU 0510-01	9 ± 1.5	1.63	1.91	43 ± 5	3840 ± 10
PESU 0510-02	10 ± 1	1.56	1.97	41 ± 8	3310 ± 515
PESU 0510-03	10 ± 0.5	1.45	1.77	26 ± 4	± *
PESU 0510-04	10 ± 0.5	1.39	1.80	23 ± 6	± *

* not determined.

**Table 6:** Heat shrinkage (MD) of selected nonwovens.

Sample	Heat Shrinkage in MD	
	Mean	Stdev.
	/%	/%
PPS-01	5.1	0.3
PPS-KV1	0.4	0.2
PEEK	0.7	0.5
PESU 0510-02	3.3	0.9
PESU 0510-02-KV2	0.3	0.1

## Data Availability

The data are available upon request due to privacy restrictions. The data presented in this study are available upon request from the corresponding author. The data are not publicly available due to running project issues.

## Abbreviations

The following abbreviations are used in this manuscript:

PFAS	Per- and polyfluoroalkyl substances
ECHA	European Chemicals Agency
PESU	Polyethersulfone
PEEK	Polyetheretherketone
PPS	Polyphenylene sulfide
EU	European Union
REACH	Registration, Evaluation, Authorization and Restriction of Chemicals
No	Number
PTFE	Polytetrafluorethylene
*T_g_*	Glass transition temperature
*T_m_*	Melting Temperature
HPP	High-performance polymers
PEI	Polyetherimide
HT	High temperature
PET	Polyethylen terephthalate
PLA	Polylactic acid
PPEK	Poly(phthalazinone ether ketone)
PAES	Polyarylthersulfone
PSU	Polysulfone
PPSU	Polyphenylene sulfone
*M_w_*	Molar mass weight average
*M_n_*	Molar mass number average
SEC	Size exclusion chromatography
DMAc	Dimethylacetamide
PMMA	Poly(methyl methacrylate)
std.	standard
WAXS	Wide-Angle X-Ray Scattering
DSC	Differential Scanning Calorimetry
MVR	Melt Volume Rate
*ΔH_m_*	Melt enthalpy
*L*	Length
*D*	Diameter
*hpi*	Holes per inch
SAH	Secondary Air Heater
IR	Infrared
DCD	Die collector distance
CD	Cross direction
SEM	Scanning electron microscopy
DIN	Deutsches Institut für Normung e. V
EN	Europäische Norm/European Standard
ISO	International Organization for Standardization
*σ_m_*	Tensile strength
*ε_m_*	Elongation at break
*ε_B_*	Elongation at peak force
E	Elastic modulus
MD	Machine direction
*G*′	Storage modulus
*G*″	Loss modulus
*χ_c_*	Degree of crystallinity
stdev	Standard deviation

## Appendix A

### Appendix A.1. Processability Analysis of Polyaryethersulfones

Figure A1 shows the temperature-dependent complex shear viscosity of all screened polyarylethersulfones.

### Appendix A.2. Nonwoven Characterization

Supplementary to the micrographs of the fabrics resulting from each process setting and material at 500-times magnification shown in Figure 6 (Section 3.3), further micrographs in 100-times magnification are shown in Figure A2.

### Appendix A.3. Statistical Evaluation of the Fiber Diameter Distributions

The diameter distribution data (diameter fraction upper-limit, absolute quantity, relative quantity, and cumulative relative quantity) were analyzed applying the Pearson’s chi-square test of homogeneity to substantiate the statistical validity of the data underlying the distribution curves. The results are shown in Table A1.

**Table A1:** Pearson’s chi-square test of homogeneity.

Group	X^2^	Degrees of Freedom	*p*-Value	Cramérs V
PPS-01–03	148.862	324	<0.01	0.3909
PESU-0510-01-04	46.993	432	<0.01	0.0566

For PPS, the three distributions differ significantly (*p*-value); the effect size (Cramérs V) is relatively large. Following this, the impact of the process temperature as a varied key parameter is significant for the fiber formation within PPS. For PESU-0510, the distributions are also statistically distinguishable. However, the effect size of the overall comparison is small. Thus, the statement “*the peaks of PESU 0510 are shifted to lower fiber diameters but don’t differ significantly among each other and thus with the increasing temperature*” is statistically valid.
